# Unilateral Hearing Loss: Listening Ability, Social Participation and Adaptive Strategies to Challenges in the Real World

**DOI:** 10.3390/audiolres16050133

**Published:** 2026-09-07

**Authors:** Joaquin T. Valderrama, Colin Barbier, Paola Incerti, Jorge Mejia, Melanie Ferguson

**Affiliations:** 1 Department of Signal Theory, Telematics and Communications, University of Granada, 18014 Granada, Spain; 2 Research Centre for Information and Communications Technologies (CITIC-UGR), University of Granada, 18014 Granada, Spain; 3 National Acoustic Laboratories, Sydney, NSW 2113, Australia; cquba@dtu.dk (C.B.); paola.incerti@nal.gov.au (P.I.); geogem245@gmail.com (J.M.); melanie.ferguson@curtin.edu.au (M.F.); 4 Department of Linguistics, Macquarie University, Sydney, NSW 2113, Australia; 5 Hearing Systems, Department of Health Technology, Technical University of Denmark, 2800 Copenhagen, Denmark; 6 Copenhagen Hearing and Balance Center, Rigshospitalet, 2100 Copenhagen, Denmark; 7 School of Computing, Macquarie University, Sydney, NSW 2113, Australia; 8 Curtin School of Allied Health, Faculty of Health Sciences, Curtin University, Perth, WA 6102, Australia

**Keywords:** unilateral hearing loss (UHL), speech-in-noise, functional hearing, ecological assessment, head orientation, reaction time, social participation, multimodal assessment

## Abstract

**Background**: Unilateral hearing loss (UHL) is associated with speech-in-noise difficulties, impaired spatial hearing, and reduced social participation, yet conventional audiological assessments may not capture these real-world consequences. This study characterised the multidimensional functional impact of UHL using an ecologically oriented test battery and explored whether accessible behavioural and self-reported measures accounted for individual differences in speech-in-noise comprehension. **Methods**: Fifteen adults with moderate to profound sensorineural UHL and sixteen adults with normal hearing completed standardised questionnaires assessing hearing disability and social participation, an auditory reaction-time task in quiet and noise, the National Acoustic Laboratories Dynamic Conversations Test, head-orientation tracking, and post-task self-reports. Group differences were assessed using mixed-effects models and independent-samples *t*-tests. An exploratory regression examined speech-in-noise performance within the UHL group. **Results**: Participants with UHL reported greater hearing difficulties and social participation restrictions, and showed modestly poorer speech-in-noise comprehension although the UHL–NH difference in speech-in-noise comprehension was no longer statistically significant after adjustment for age. Reaction times were significantly longer in noise than in quiet but did not differ significantly between hearing groups. The UHL group also showed a significantly more pronounced head orientation towards the poorer-hearing side and greater within-participant head-orientation variability. An exploratory model combining reaction time in noise with three post-task self-report items showed strong apparent in-sample fit (adjusted $R^{2}$ = 0.71). However, internal cross-validation of the complete model-selection procedure yielded substantially lower predictive performance ($R_{\mathrm{CV}}^{2}$ = 0.19). **Conclusions**: UHL is associated with differences across complementary perceptual, behavioural, and self-reported dimensions of everyday communication. Multimodal functional assessment may provide information beyond conventional audiometry, although the exploratory model requires validation in larger, age-matched independent cohorts.

## 1. Introduction

Approximately 7.2% of adults in a population-based US sample had unilateral hearing loss (UHL) [1]. UHL encompasses a range of hearing-loss severities, whereas severe-to-profound unilateral loss is commonly referred to as single-sided deafness (SSD). UHL is associated with functional difficulties that extend beyond reduced audibility in the poorer ear. Although definitions, degrees of hearing loss, and aetiologies vary across studies [1], a consistent body of evidence demonstrates that reduced access to bilateral auditory input compromises speech understanding and spatial hearing, particularly in acoustically complex listening environments. Experimental studies have shown that binaural hearing provides substantial advantages for speech recognition in the presence of competing talkers and for sound localisation, advantages that are largely lost when listeners rely on a single ear [2]. These findings are supported by broader reviews describing how the loss of binaural cues disrupts spatial hearing, speech perception in noise, and the ability to segregate competing sound sources [3].

Clinical studies consistently demonstrate that the auditory deficits associated with UHL translate into reduced functional hearing during everyday communication. Adults with acquired UHL frequently report problems understanding speech in background noise, following conversations involving multiple talkers, localising unseen sound sources, and increased listening effort [4]. Similarly, adults relying on a single normally hearing ear exhibit poorer speech, spatial, and sound-quality abilities than listeners with bilateral normal hearing, despite normal hearing sensitivity in the better ear [5]. Perceived hearing handicap has also been documented following acquired unilateral sensorineural hearing loss [6], while substantial inter-individual variability in self-perceived disability indicates that audiometric thresholds alone are insufficient to predict the functional impact of UHL [7]. Furthermore, Wie et al. [8] reported that most adults with profound UHL experienced difficulties communicating in noisy environments and commonly adopted compensatory behaviours such as head positioning and speechreading to facilitate communication.

The consequences of UHL extend beyond auditory performance to affect quality of life and social participation. Qualitative and patient-reported studies describe reduced confidence in communication, withdrawal from challenging listening situations, increased fatigue, and concerns regarding future hearing deterioration [8,9]. More recently, Apa et al. [10] reported poorer social functioning and psychological well-being in a case–control study of working adults with single-sided deafness compared with normal-hearing controls. Likewise, difficulties with speech discrimination and sound localisation have been associated with poorer health-related quality of life in adults with profound UHL following cerebellopontine-angle surgery [11]. Although these surgical populations may experience additional factors beyond hearing loss itself, the findings further illustrate the broader functional consequences associated with unilateral hearing impairment.

Building upon this body of evidence, Galloway et al. [12] used semi-structured interviews and open-ended surveys to characterise the lived experiences of adults with UHL. In addition to confirming persistent difficulties with speech understanding in noise, sound localisation, and listening fatigue, participants described adopting a range of compensatory strategies, including repositioning themselves relative to talkers, turning the better-hearing ear towards the sound source, relying on visual speech cues, and actively seeking quieter listening environments. These observations suggest that behavioural adaptation constitutes an important component of everyday communication with UHL.

However, despite extensive evidence describing the perceptual, behavioural, and psychosocial consequences of UHL, these domains have largely been investigated in isolation. Relatively few studies have simultaneously examined objective speech perception, behavioural adaptation during communication, and patient-reported outcomes within the same experimental framework. Consequently, important questions remain regarding how these complementary dimensions interact during everyday communication. Addressing this challenge requires assessment approaches that more closely reflect the complexity of real-world listening situations rather than relying solely on conventional audiological measures.

In hearing science, ecological validity refers to the degree to which research findings reflect real-life hearing-related function, activity, or participation [13,14]. Conventional audiological tests provide essential information about auditory sensitivity and speech recognition under standardised conditions, but they are typically based on relatively brief and constrained listening tasks that capture only part of the perceptual, cognitive, spatial, and behavioural demands encountered in daily communication. Increasing ecological validity therefore requires not only realistic acoustic environments but also outcome measures and experimental tasks that capture the perceptual, cognitive, and behavioural demands of everyday communication [13].

Because everyday communication involves perceptual, cognitive, behavioural, and subjective components, no single outcome measure can fully characterise the functional impact of UHL. To address this challenge, the present study adopted a multidimensional and ecologically oriented assessment framework integrating complementary outcome measures that capture different aspects of functional hearing. Standardised questionnaires assessed participants’ long-term perception of hearing ability and social participation; reaction time provided an objective behavioural measure of response speed during an auditory task; the National Acoustic Laboratories–Dynamic Conversations Test (NAL-DCT) evaluated speech comprehension during realistic multi-talker conversations in noise; head-orientation tracking quantified head-orientation behaviour during communication; and a brief self-report survey captured participants’ immediate listening experiences following each conversation. Collectively, these complementary measures were selected to characterise functional hearing beyond speech recognition performance alone [15].

Accordingly, the objectives of this study were twofold: (1) to characterise the functional impact of UHL using a multidimensional and ecologically oriented assessment framework that evaluated speech-in-noise comprehension, auditory reaction time, head-orientation behaviour, and self-reported hearing experiences; (2) to investigate whether a small set of accessible behavioural and self-reported measures could account for individual differences in functional speech communication in adults with UHL. This multidimensional framework aligns with contemporary recommendations that functional hearing should be evaluated across complementary domains relevant to everyday communication rather than through auditory sensitivity alone [13]. Ultimately, integrating complementary behavioural and self-reported outcome measures may facilitate the development of efficient and personalised approaches for assessing functional hearing difficulties in clinical practice.

## 2. Materials and Methods

### 2.1. Participants

Participants were recruited from the National Acoustic Laboratories (NAL, Sydney, Australia) Research Participants Database (a register of people who have given their consent to be invited to participate in NAL research); Hearing Australia clients (a government-funded hearing service provider); local hearing clinics; staff and students from NAL and Macquarie University (Sydney, Australia); and through advertisements on social media and the Macquarie University campus. The study involved participants with normal hearing (NH) and unilateral hearing loss (UHL).

Participants in both groups were required to be at least 18 years old and native English speakers. Cognitive status was not formally assessed as part of the study. NH was defined as a four-frequency air-conduction pure-tone average (PTA; 0.5, 1, 2, and 4 kHz) of ≤25 dB HL in both ears. Various definitions of UHL exist, often specifying different hearing thresholds for the poorer ear [1]. In the present study, UHL was defined as normal hearing in the better ear (PTA ≤ 25 dB HL) and sensorineural hearing loss of at least moderate degree in the poorer ear (PTA ≥ 40 dB HL).

Air- and bone-conduction pure-tone audiometry was measured using the AC40 clinical audiometer (Interacoustics A/S, Middelfart, Denmark) in a soundproof booth. Air-conduction thresholds were estimated at 0.125, 0.25, 0.5, 1, 1.6, 2, 4, and 8 kHz; and bone-conduction thresholds at octave frequencies from 0.25 to 4 kHz.

Sixteen NH (12 females, 18–70 years, mean ± SD = 37.2 ± 19.5 years) and 15 UHL (5 females, 25–75 years, mean ± SD = 50.9 ± 15.6 years) participants met the inclusion criteria and completed the study. Participants with UHL were significantly older than NH participants (mean difference = 13.7 years, 95% CI [0.7–26.6], Welch’s *t*(28.30) = 2.16, *p* = 0.039, Cohen’s *d* = 0.75). Given this between-group age difference and the potential influence of age on auditory and behavioural outcomes, age was included as a covariate in subsequent sensitivity analyses.

In the UHL group, four participants acquired their hearing loss during the first four years of life, four between 4 and 10 years of age, and the remaining seven during adulthood. Accordingly, the duration of UHL at the time of testing varied widely across participants, ranging from approximately 1 to 65 years, with three participants presenting congenital UHL. All UHL participants presented sensorineural hearing loss. Ten UHL participants presented hearing loss in the left ear. The origin of their hearing loss varied among participants, including mumps, meningitis, measles, influenza, Ménière’s disease, acoustic neuroma, or damaged auditory nerve. Eleven UHL participants presented profound hearing loss (PTA greater than 90 dB HL), three had severe hearing loss (PTA between 71 and 90 dB HL), and one of them had moderate hearing loss (PTA between 40 and 55 dB HL).

Participants’ demographic information, individual hearing profiles, age at onset and estimated duration of UHL, and air- and bone-conducted pure-tone audiometry hearing thresholds are available in Supplementary S1 of the Online Supplemental Materials.

### 2.2. Experimental Protocol and Data Analysis

**Figure 1:**
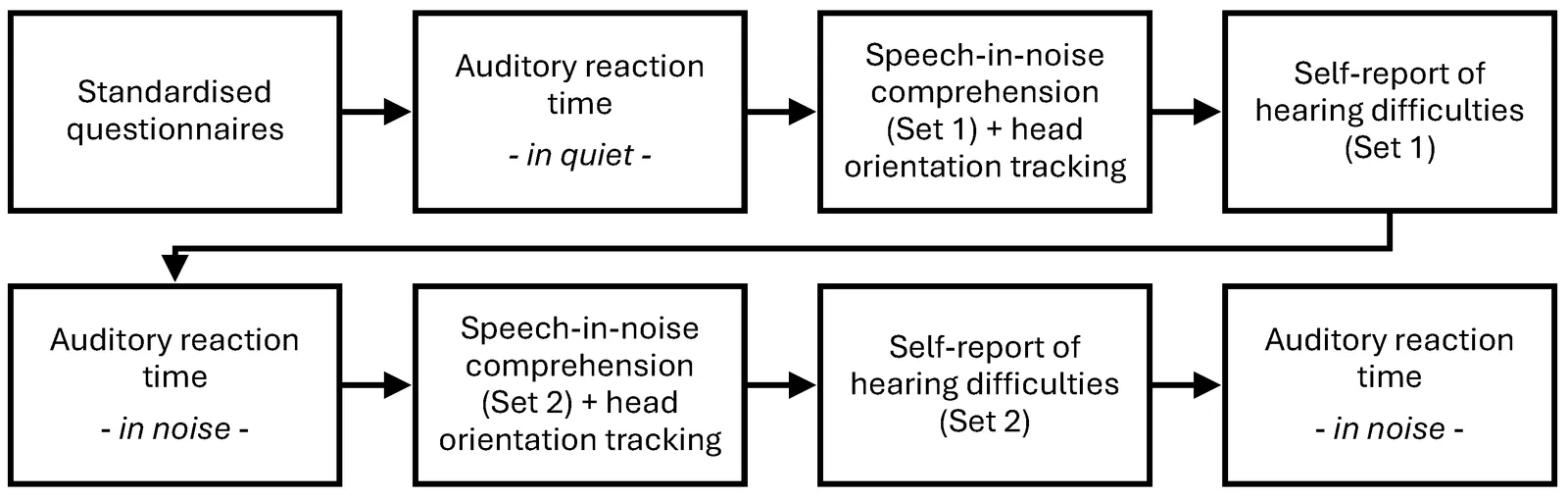
Sequence of assessments and tasks in the experimental protocol, including auditory reaction-time testing, speech-in-noise comprehension, head-orientation tracking, and self-report of hearing difficulties.

Participants attended a single, three-hour appointment at the Australian Hearing Hub (Sydney, Australia). Experimental testing was conducted in a fully anechoic chamber, using an array of 41 speakers spherically distributed in five rows. Figure 1 outlines the sequence of the experimental protocol, which included assessments of the participants’ hearing experience using standardised questionnaires, measurements of auditory reaction time during speech-listening tasks performed in quiet and in background noise, head orientation tracking during a speech-in-noise comprehension task, and self-perceived evaluations of hearing difficulties. Information regarding participants’ habitual use of hearing devices was not systematically collected; however, all experimental testing was conducted unaided, with no hearing devices used during the assessments. The sections below detail the testing protocols and data analysis for these assessments. Data analyses were performed in MATLAB (R2024a, The MathWorks Inc., Natick, MA, USA), using functions from the *Statistics and Machine Learning* toolbox. Data normality was evaluated via the Lilliefors statistical test, which is used to assess whether a sample comes from a normally distributed population when the population mean and variance are unknown [16]. Supplementary S2 of the Online Supplemental Materials provides raw data and custom MATLAB scripts that re-generate figures and perform the statistical analyses presented in the Results section (Section 3).

**Standardised questionnaires**

Participants’ self-perceived hearing difficulties and social participation restrictions were measured at home using an online survey prior to attending the test session. These measures comprised the short form of the Speech, Spatial and Qualities of Hearing Scale (SSQ12) [17] and the Social Participation Restrictions Questionnaire (SPaRQ) [18,19]. The SSQ12 assesses individuals’ perceived hearing abilities in everyday life, providing insights into the practical impact of hearing on daily interactions and environmental awareness. It measures three key dimensions: (1) *speech hearing*—to assess the ability to understand speech in various listening conditions, such as in noise or in groups; (2) *spatial hearing*—to assess perception of sound direction, distance, and movement, helping to gauge how well individuals can locate sounds in their environment; (3) *qualities of hearing*—to capture sound clarity, naturalness, and the ability to distinguish between sounds, reflecting the overall quality of auditory experience. A global SSQ12 score was also calculated by averaging responses across all 12 items. The SPaRQ evaluates how hearing difficulties impact a person’s social engagement and sense of inclusion. It measures restrictions in social participation through two main subscales: (1) *social behaviour*—to assess the extent to which hearing challenges affect behaviours in social settings, such as joining conversations or participating in group activities; (2) *social perceptions*—to evaluate the emotional and psychological responses to hearing difficulties in social contexts, including feelings of frustration, embarrassment, or isolation. Both measures were scored using an 11-point visual analogue scale ranging from 0 to 10, where higher scores on the SSQ12 indicate fewer difficulties, while higher scores on the SPaRQ reflect greater social participation restrictions. The scores reported by NH and UHL participants were statistically compared via two-sample *t*-tests. These comparisons were planned as part of the multidimensional characterisation of UHL. To account for multiple comparisons, *p*-values for the four SSQ12 outcomes (speech, spatial, qualities, and global scores) and the two SPaRQ subscales were jointly adjusted using the Benjamini–Hochberg false discovery rate (FDR) procedure.

**Auditory reaction time**

Auditory reaction time (RT) was measured using a single-task paradigm performed under quiet and noisy listening conditions. Participants were presented a sequence of random monosyllabic digits (i.e., those from 0, pronounced as /oh/, to 10, excluding 7) every 1.5 s, and were instructed to press a key as soon as they heard two consecutive ascending digits (i.e., an event), such as 4 followed by 5. Test duration was 2 min per run, and a total of 8 events were presented per run. Performance was quantified as the reaction time associated with each correct detection. Reaction time was interpreted as an objective behavioural measure of response speed rather than as a direct or process-specific measure of listening effort. Digits were presented at 72 dB SPL from two speakers situated in front of the participant, at a $\pm 22.5^{\circ }$ angle. This relatively small spatial separation was selected to resemble the angular separation commonly encountered between conversational partners positioned in front of the listener while avoiding unrealistically large spatial cues. Noise consisted of 8-talker babble noise, delivered from the array of 41 speakers. Three repetitions were conducted at different time points of the experimental protocol, with the first conducted in quiet and the remaining two at a signal-to-noise ratio (SNR) of 0 dB. Since RT scores were positive and right-skewed, the effects of Background (i.e., quiet vs. noise) and Hearing (NH vs. UHL) on RT were simultaneously characterised using a generalised linear mixed-effects (GLME) model [20], which accounts for repeated observations and can accommodate missing and unbalanced data [21]. In this model, reaction time was treated as the dependent variable, Background and Hearing as fixed effects, and participant was included as a random intercept to account for within-participant dependence across repeated observations. A Gamma distribution with a log link function was used, and the model was fitted using the Laplace approximation. Fixed-effect coefficients were exponentiated to facilitate interpretation, with percentage changes in reaction time calculated as $100\times [e^{\beta }-1]$.

**Speech-in-noise comprehension**

This was evaluated using the National Acoustic Laboratories Dynamic Conversations Test (NAL-DCT), which was specifically developed to assess speech comprehension under listening conditions that reproduce key features of everyday communication more closely than conventional sentence-recognition tests, including continuous conversation, multiple talkers, spatially distributed speech sources, and realistic background noise [15,22]. The test used background noise resembling a busy cafeteria, sourced from the Ambisonics Recordings of Typical Environments (ARTE) database [23], played at 72 dB SPL through an array of 41 speakers. Target speech featured a conversation between two Australian English speakers, and its material was obtained from the International English Language Testing System (IELTS) English test. The two talkers were presented from speakers positioned at 45° and 22.5° azimuth relative to the participants’ worse and better hearing ears, respectively. This asymmetrical configuration was deliberately designed to allow participants to orient their heads naturally during the conversation, as they would when interacting with different speakers in everyday face-to-face communication. This approach was intended to enhance the sensitivity of the paradigm to functional communication strategies associated with unilateral hearing loss. For participants with normal hearing, speaker positions were randomised between the two possible configurations (−22.5° and 45°, or 22.5° and −45°). Speech was delivered at 0 dB SNR to approximate a challenging everyday speech-in-noise condition. Participants listened to six conversations in noise (3–4 min each). Each conversation was split into three parts, and after each part they were asked 3–4 multiple-choice questions that assessed their comprehension, with a total of 10 questions per conversation, and scores expressed as percentage correct. The first three conversations were included in Set 1 of the experimental protocol, and the remaining three in Set 2. As NAL-DCT scores followed a normal distribution, comprehension performance was analysed using a linear mixed-effects model (LME) [24], with NAL-DCT score as the dependent variable, hearing group (NH vs. UHL) and conversation (Run 1–6) as fixed effects, and participant as a random intercept. Conversation was included as a categorical factor to account for systematic differences in difficulty across the six NAL-DCT conversations.

**Head orientation tracking**

To complement speech-comprehension performance, head orientation was simultaneously recorded throughout the NAL-DCT to characterise head-orientation behaviour during communication. Head movements were measured during the NAL-DCT speech-in-noise comprehension task using the InterSense InertiaCube^®^ 4 (Thales Defense & Security, Inc., Billerica, MA, USA)—a compact inertial measurement unit that tracks three-dimensional orientation and motion with accelerometers, gyroscopes, and magnetometers. To provide a controlled visual cue to the active talker’s location, blue LEDs on the loudspeakers indicated the real-time active talker’s position [25]. The head tracker was secured on participants’ heads with a headband, and MATLAB software synchronised the audio stimuli with head movement data acquisition, sampled at 44,100 Hz. Head movement was analysed with a histogram showing the percentage of time spent at each head orientation angle, with positive angles indicating the better hearing ear. Signal processing steps included (1) downsampling the azimuth orientation data to 4410 Hz, (2) inverting the orientation data for UHL participants with their worse ear on the right, (3) removing the first and last 5 s to exclude non-task movements, and (4) creating a histogram with a 2.5° resolution. After excluding 23 NH recordings and 15 UHL recordings because of technical or calibration problems, 148 of the 186 available recordings remained for analysis. One participant from each group had no valid head-tracking recordings and therefore did not contribute to the head-orientation analyses, resulting in 15 NH and 14 UHL participants with analysable data. For each participant, mean head orientation was calculated by averaging the mean orientations across all usable NAL-DCT recordings. Within-participant head-orientation variability was quantified by calculating the standard deviation of head orientation within each usable recording and then averaging these standard deviations across recordings. Between-group differences in both outcomes were assessed using Welch’s independent-samples *t*-tests. A sensitivity analysis repeated these comparisons after restricting the sample to participants with at least five of the six recordings available.

**Self-report of hearing difficulties**

After completing each set of three conversations in the NAL-DCT speech-in-noise comprehension task, participants rated various aspects of their hearing experience using a 10-item survey: (1) *How noisy is it?* (2) *How much of the talker’s speech can you understand?* (3) *How much does the noise interfere with your ability to listen to the talker/s?* (4) *Does your hearing restrict your ability to listen to the talker/s?* (5) *How annoying is this listening environment?* (6) *How frustrated do you feel?* (7) *How demanding is it to listen to the talker?* (8) *How mentally tired do you feel?* (9) *When the talking moves from one speaker to the other, do you miss the start of what is being said?* (10) *How confident are you that you can tell where the talker is coming from?* Responses were recorded on five-level Likert scales, with higher scores indicating greater hearing difficulties, except for questions 2 and 10, which were reverse-scored. Anchors for each response level of every question are provided in Supplementary S2 of the Online Supplemental Materials. For each participant, responses to each item were averaged across the two NAL-DCT sets, yielding one score per item. The item-level analyses were exploratory. Group differences for each item were assessed using independent-samples *t*-tests, with the resulting *p*-values jointly adjusted across the 10 items using the Benjamini–Hochberg false discovery rate (FDR) procedure. Given the five-level ordinal response format, Wilcoxon rank-sum tests were additionally performed as a non-parametric sensitivity analysis, with the resulting *p*-values likewise adjusted using the Benjamini–Hochberg FDR procedure.

**Exploratory regression model**

To address the second research objective, we conducted an exploratory analysis to determine whether a combination of reaction time and self-reported hearing difficulties could account for individual differences in speech-in-noise comprehension within the UHL group. A total of 176 candidate linear regression models were evaluated: one model containing reaction time in noise alone, and 175 models containing reaction time together with a self-report composite derived from all possible combinations of one, two, or three items from the 10-item survey. For combinations containing more than one questionnaire item, item scores were averaged to form a single self-report predictor. The number of questionnaire items included in the composite was restricted to a maximum of three to achieve a pragmatic balance between explanatory performance and parsimony. The model with the highest adjusted $R^{2}$ was retained as the best-fitting exploratory model. Predictors in the selected model were subsequently standardised (z-scores) to facilitate interpretation and comparison of the regression coefficients.

To assess the optimism introduced by this data-driven model-selection procedure, internal validation was performed using leave-one-out cross-validation (LOOCV) of the complete model-selection process. In each fold, one participant was held out, all 176 candidate models were re-evaluated using the remaining 14 participants, and the model with the highest adjusted $R^{2}$ in the training sample was used to predict the NAL-DCT score of the held-out participant. Cross-validated performance was quantified using the cross-validated coefficient of determination ($R_{\mathrm{CV}}^{2}$), root-mean-square error (RMSE), and mean absolute error (MAE). Given the small sample and absence of external validation, the analysis was regarded as hypothesis-generating and proof-of-concept rather than as a validated predictive model.

## 3. Results

### 3.1. Standardised Questionnaires

**Figure 2:**
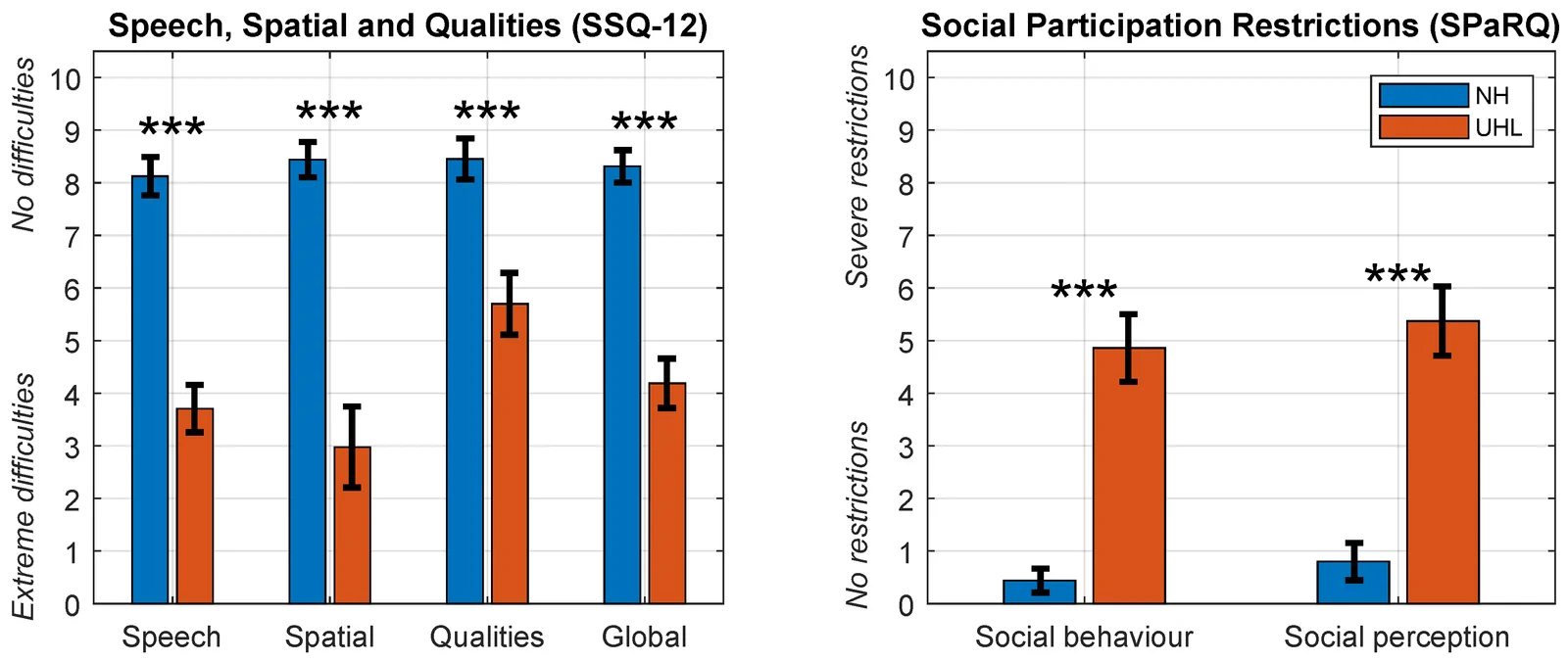
Mean and standard error of the mean (error bars) for the global score and subscales of the short form of the Speech, Spatial and Qualities questionnaire (SSQ12, left panel) and the Social Participation Restrictions Questionnaire (SPaRQ, right panel) for the normal hearing (NH) and unilateral hearing loss (UHL) groups. *** FDR-adjusted *p* < 0.001.

Figure 2 presents the mean and standard error of the mean for the global scores and subscales of the SSQ12 and the SPaRQ for the NH and UHL groups. NH individuals reported minimal hearing difficulties and social participation restrictions. In contrast, UHL participants reported greater challenges with speech understanding (SSQ12, Speech); sound direction, localisation, and distance perception (SSQ12, Spatial); hearing clarity, naturalness, and effort (SSQ12, Qualities); performing tasks in social contexts due to hearing issues (SPaRQ, Social behaviour); and emotional comfort and confidence (SPaRQ, Social perception). All between-group differences shown in Figure 2 remained statistically significant after Benjamini–Hochberg FDR correction (all FDR-adjusted *p* < 0.001). Full results of the multiple-comparison correction are provided in Supplementary S3 (Section S3.1.1) of the Online Supplemental Materials.

### 3.2. Auditory Reaction Time

**Figure 3:**
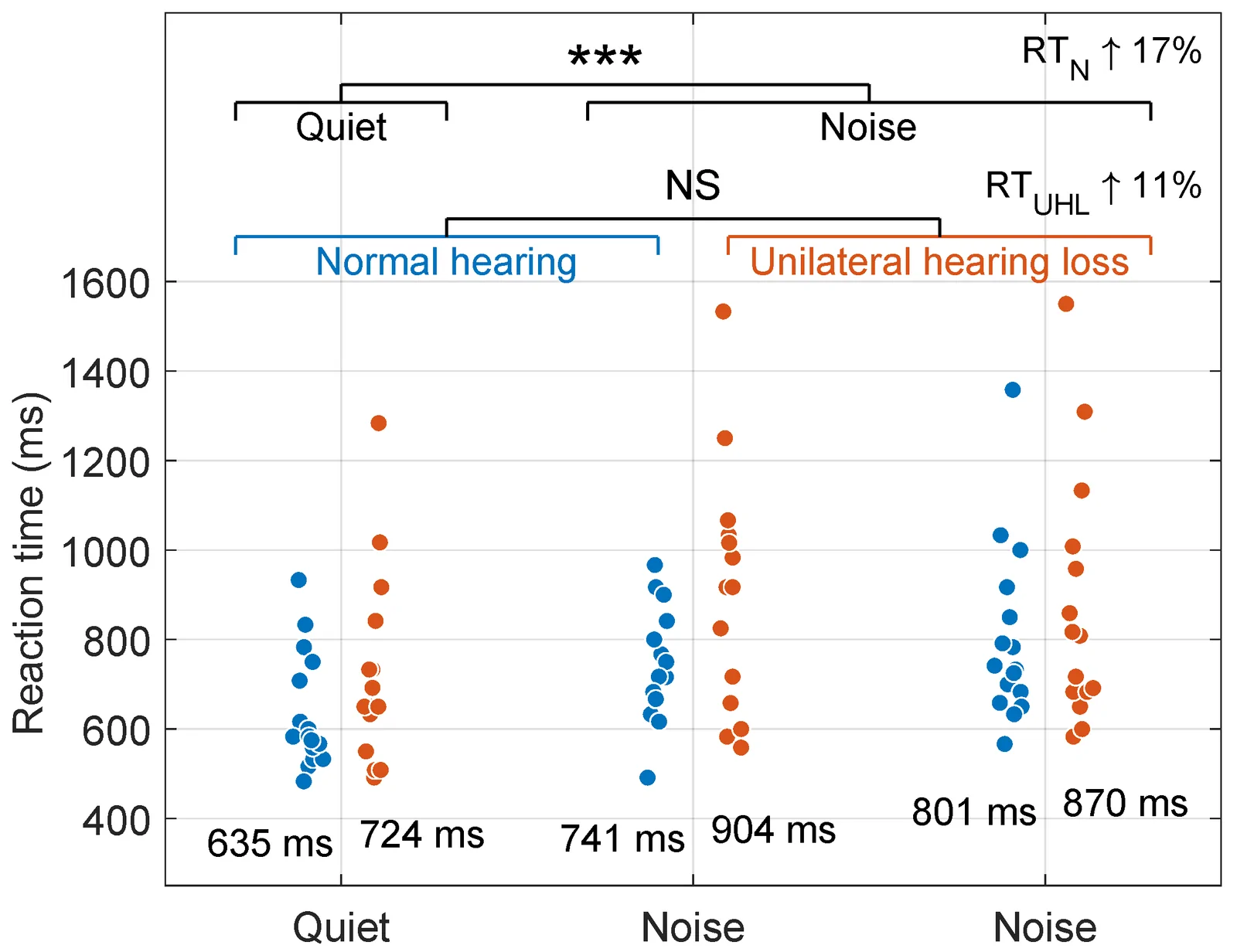
Median reaction times (RTs, ms) for each participant in quiet (Q) and noise (N) conditions for the normal-hearing (NH, blue) and unilateral hearing loss (UHL, orange) groups. The mean of participants’ median reaction times is displayed below each distribution. Upward arrows indicate model-estimated increases in RT from the generalised linear mixed-effects model reported in Table 1: approximately 17% in noise relative to quiet and approximately 11% in the UHL group relative to the NH group. The noise-related increase was statistically significant (*** *p* < 0.001), whereas the UHL–NH difference was not significant (NS).

Figure 3 shows the median reaction times for each participant in quiet and noise. The group mean of participants’ median reaction times is shown below each distribution. The effects of listening condition (quiet vs. noise) and hearing group (NH vs. UHL), estimated using the generalised linear mixed-effects model, are shown in Table 1. Listening condition had a significant effect on reaction time (β=−0.158, SE = 0.026, *p* < 0.001), with model-estimated reaction times approximately 17% longer in noise than in quiet (calculated as $100\times [e^{-\beta_{Quiet}}-1]$). Although model-estimated reaction times were approximately 11% longer for participants with UHL than for those with NH (i.e., $100\times [e^{\beta_{UHL}}-1]$), the effect of hearing group was not statistically significant (β=0.104, SE = 0.070, *p* = 0.136).

An age-adjusted sensitivity analysis yielded the same overall pattern of results: listening condition remained significantly associated with reaction time (β=−0.158, SE = 0.026, *p* < 0.001), whereas neither hearing group (β=0.122, SE = 0.075, *p* = 0.105) nor age (β=−0.0012, SE = 0.0020, *p* = 0.543) showed a statistically significant effect. Full results of this sensitivity analysis are provided in Supplementary S3 (Section S3.2.1) of the Online Supplemental Materials.

### 3.3. Speech-in-Noise Comprehension

**Figure 4:**
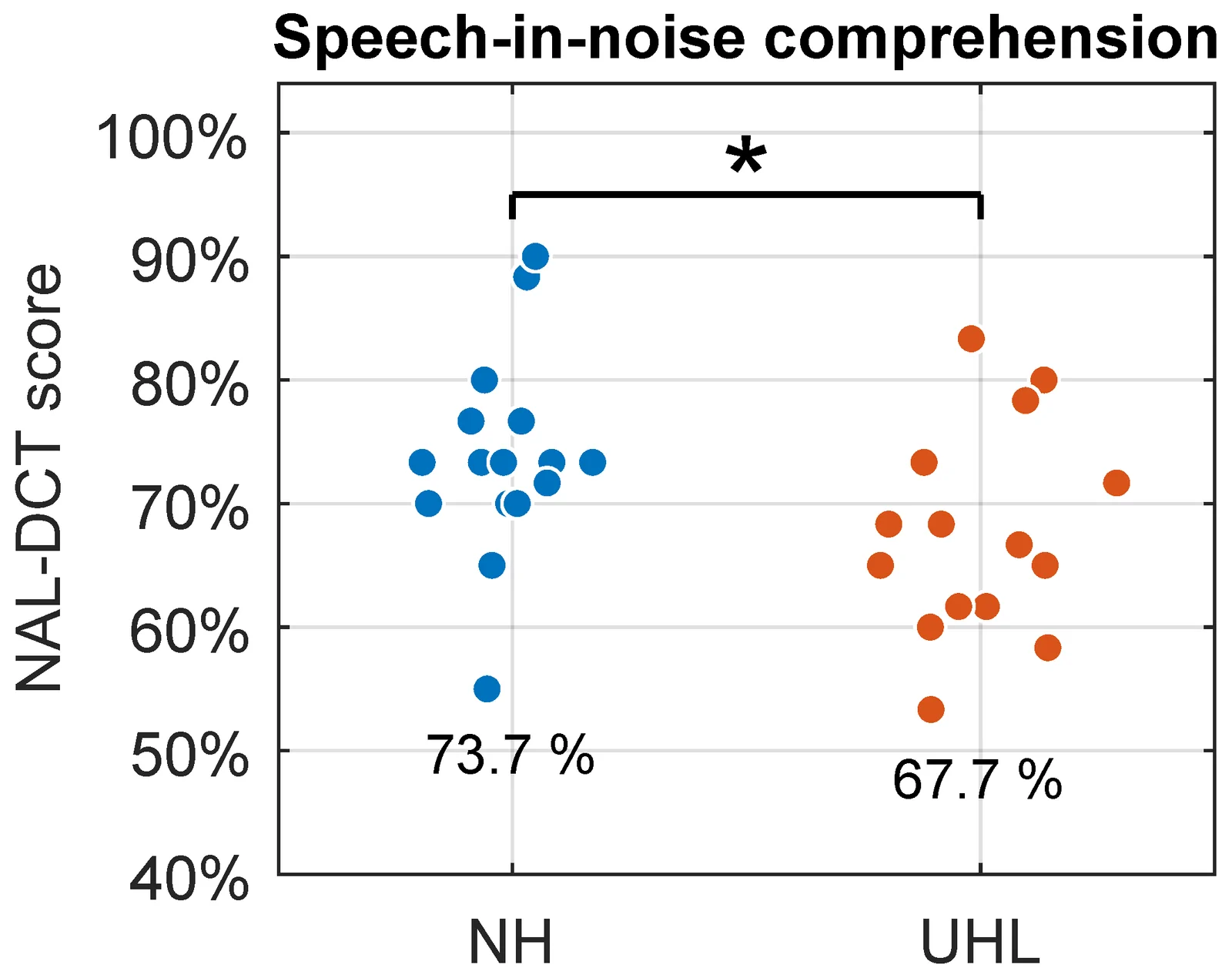
Speech-in-noise comprehension in terms of the NAL-DCT score (% correct) for the normal hearing (NH) and unilateral hearing loss (UHL) groups. The significance marker represents the hearing-group effect from the linear mixed-effects model reported in Table 2. * *p* < 0.05.

Figure 4 shows the mean NAL-DCT scores (% correct) across the six conversations, which were 73.7% for the NH group and 67.7% for the UHL group. The linear mixed-effects model presented in Table 2 showed a significant effect of hearing group, with NAL-DCT scores estimated to be 6.08 percentage points lower in participants with UHL than in those with NH (β=−6.08, SE = 2.99, *p* = 0.043). There was also a significant overall effect of conversation (F(5,179) = 22.14, *p* < 0.001), indicating differences in comprehension performance across the six conversations.

An age-adjusted sensitivity analysis yielded a similar estimated between-group difference in magnitude, with NAL-DCT scores estimated to be 5.58 percentage points lower in participants with UHL than in those with NH. However, the hearing-group effect no longer reached statistical significance after adjustment for age (β=−5.58, SE = 3.27, *p* = 0.089). Age itself was not significantly associated with NAL-DCT performance (β=−0.037, SE = 0.088, *p* = 0.676). Full results of this sensitivity analysis are provided in Supplementary S3 (Section S3.2.2) of the Online Supplemental Materials.

### 3.4. Head Orientation

**Figure 5:**
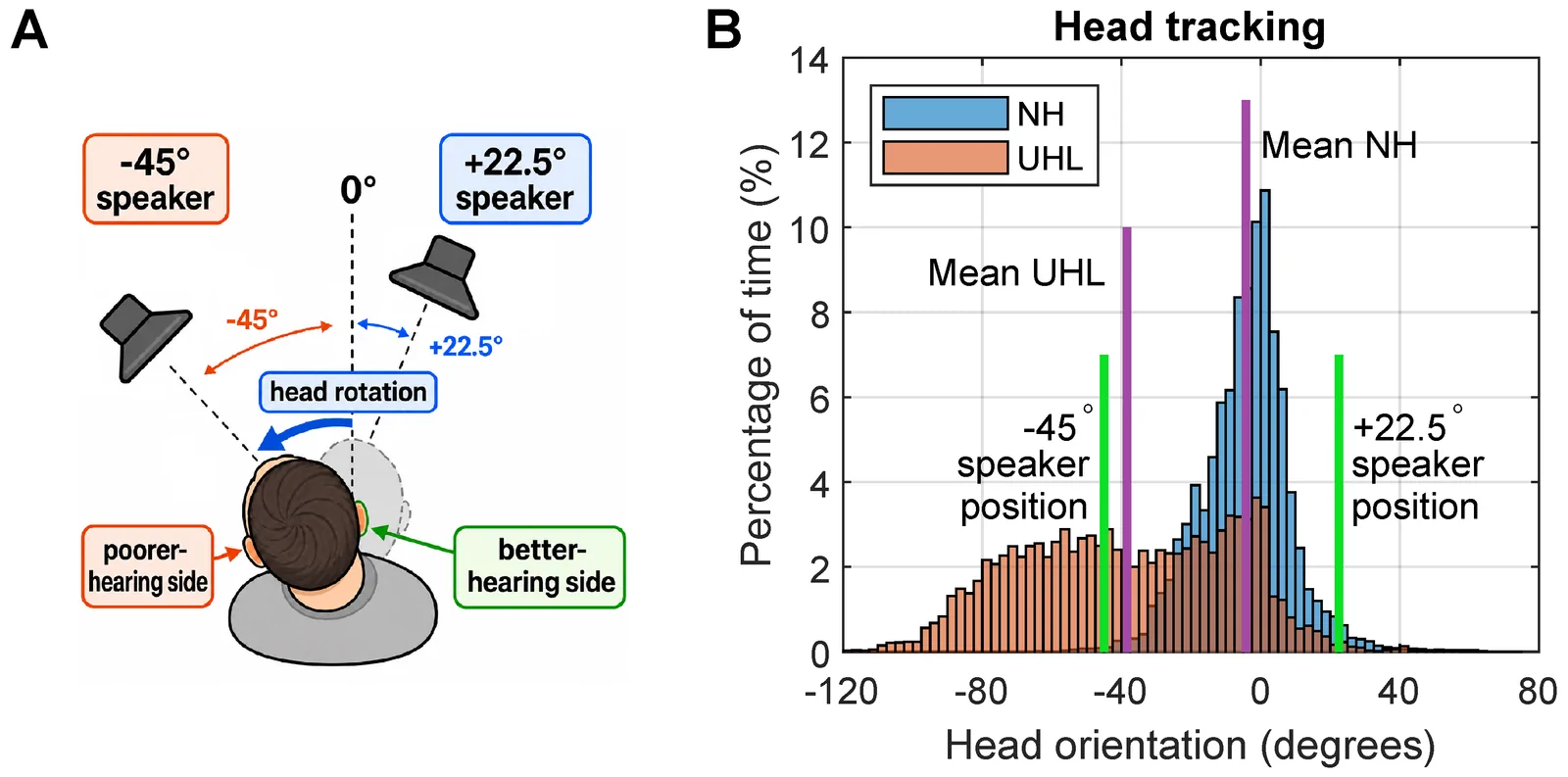
Head-orientation behaviour during the NAL-DCT. (**A**) Experimental loudspeaker configuration and head-rotation convention, with talkers positioned at $-45^{\circ }$ on the poorer-hearing side and $+22.5^{\circ }$ on the better-hearing side; the unrotated head position is shown in grey. (**B**) Combined distributions of head orientation for the normal-hearing (NH) and unilateral-hearing-loss (UHL) groups, expressed as the percentage of time spent at each azimuth angle. For UHL participants, positive angles indicate orientation towards the better-hearing side and negative angles towards the poorer-hearing side; NH data were recoded according to the corresponding loudspeaker configuration. Green vertical lines indicate the two loudspeaker positions, and purple vertical lines indicate the mean head orientation for each group (NH: $-4.3^{\circ }$; UHL: $-38.4^{\circ }$).

Figure 5 shows the experimental configuration and the combined distributions of head orientation during the NAL-DCT speech-in-noise comprehension task for the NH and UHL groups. For participants with UHL, positive angles represent orientation towards the better-hearing side, whereas negative angles represent orientation towards the poorer-hearing side; NH data were recoded according to the corresponding loudspeaker configuration. Mean head orientation was $-4.30^{\circ }$ ± $3.72^{\circ }$ in the NH group and $-38.39^{\circ }$ ± $18.55^{\circ }$ in the UHL group. Participants with UHL therefore showed a significantly more negative mean head orientation than participants with NH (UHL–NH difference = $-34.09^{\circ }$, 95% CI [$-44.93^{\circ }$, $-23.26^{\circ }$], Welch’s *t*(13.97) = −6.75, *p* < 0.001), indicating greater orientation towards the poorer-hearing side under the experimental listening configuration. Within-participant head-orientation variability was also significantly greater in the UHL group ($21.14^{\circ }$ ± $4.22^{\circ }$) than in the NH group ($10.43^{\circ }$ ± $5.12^{\circ }$; UHL–NH difference = $10.71^{\circ }$, 95% CI [$7.14^{\circ }$, $14.28^{\circ }$], Welch’s *t*(26.62) = 6.16, *p* < 0.001). A sensitivity analysis restricted to participants with at least five of the six recordings available yielded highly similar between-group differences in both mean orientation and orientation variability. Full results are provided in Supplementary S3 (Section S3.3) of the Online Supplemental Materials.

### 3.5. Self-Report of Hearing Difficulties

**Figure 6:**
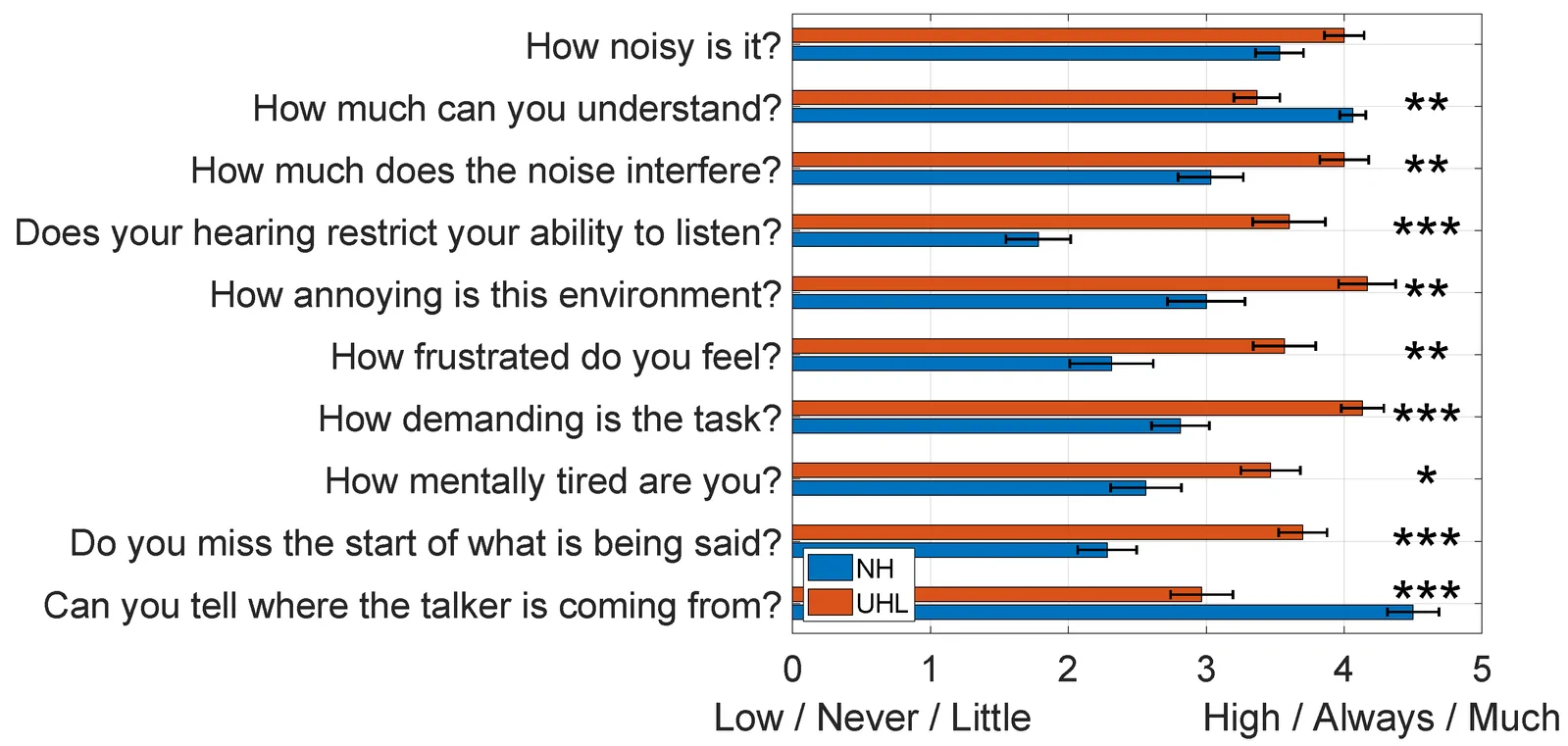
Mean and standard error of the mean (error bars) for responses to ten questions about participants’ hearing experiences during the NAL-DCT speech-in-noise comprehension test, comparing the normal hearing (NH) and unilateral hearing loss (UHL) groups. The full, original questions and anchors for each response level are provided in Supplementary S2 of the Online Supplemental Materials. * FDR-adjusted *p* < 0.05, ** FDR-adjusted *p* < 0.01, *** FDR-adjusted *p* < 0.001.

Figure 6 shows responses from the NH and UHL groups to ten questions about their hearing experience during the NAL-DCT speech-in-noise comprehension test. Following Benjamini–Hochberg FDR correction across the 10 exploratory item-level comparisons, participants with UHL reported significantly poorer outcomes than participants with NH for Q2–Q10, including reduced speech understanding, greater perceived listening demand and mental fatigue, and more difficulty with spatial hearing and following conversations (all FDR-adjusted *p* < 0.05). Perceived noisiness (Q1) did not differ significantly between groups (FDR-adjusted *p* = 0.063). A non-parametric sensitivity analysis using Wilcoxon rank-sum tests yielded the same inferential pattern after FDR correction, with Q2–Q10 remaining significant and Q1 remaining non-significant. Full results are provided in Supplementary S3 (Section S3.1.2) of the Online Supplemental Materials.

### 3.6. Exploratory Regression Model

**Figure 7:**
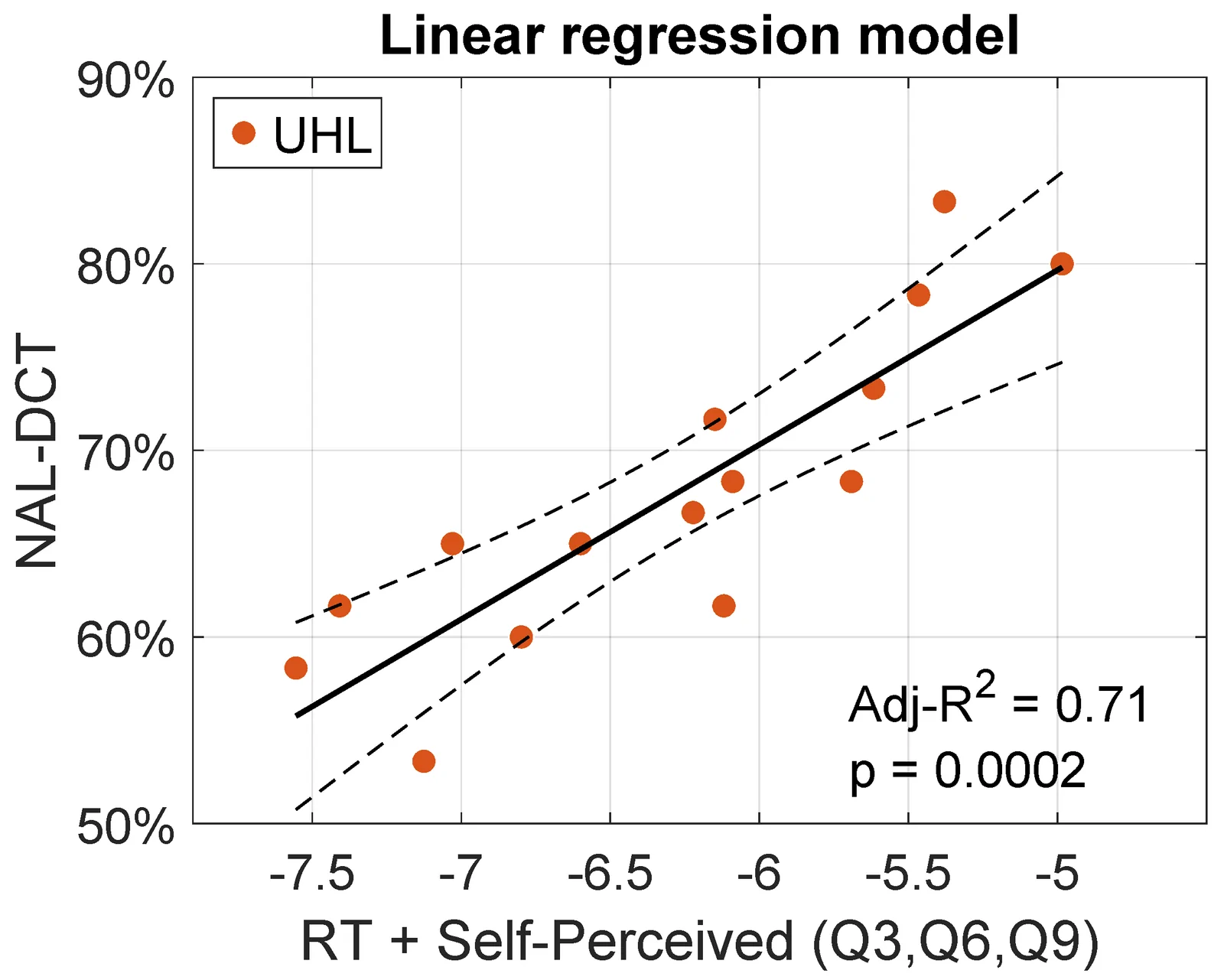
Relationship between speech-in-noise comprehension and the linear predictor obtained from the best-fitting exploratory regression model in participants with unilateral hearing loss (UHL). The y-axis shows the NAL Dynamic Conversations Test (NAL-DCT) comprehension score (% correct), whereas the x-axis represents the weighted linear combination of standardised reaction time in noise and the standardised composite score derived from three self-report items: noise interference (Q3), frustration (Q6), and missing the start of sentences (Q9). Each point represents one participant. The solid line indicates the fitted linear relationship, and dashed lines represent the 95% confidence interval. The model showed a strong apparent in-sample fit (adjusted *R*^2^ = 0.71, *p* = 0.0002).

Having identified differences across several complementary dimensions of functional listening, we next explored whether a combination of these measures could account for individual differences in speech-in-noise performance. Figure 7 shows the best-fitting exploratory linear regression model relating NAL-DCT scores in the UHL group to reaction time in noise and the mean score across three self-report items: noise interference (Q3), frustration (Q6), and missing the start of sentences (Q9). The model was statistically significant (F(2,12) = 18.1, *p* = 0.0002) and showed a strong apparent in-sample fit (adjusted *R*^2^ = 0.71). Table 3 presents the estimated coefficients of the model using normalised (z-score) input data. The standardised coefficients for reaction time and the selected self-report composite were similar in magnitude, and both effects were statistically significant.

Internal validation of the complete model-selection procedure using leave-one-out cross-validation yielded substantially lower predictive performance ($R_{\mathrm{CV}}^{2}$ = 0.19, RMSE = 7.34 percentage points, MAE = 5.82 percentage points), indicating considerable optimism in the apparent in-sample fit. Nevertheless, the Q3–Q6–Q9 composite was reselected as the best-performing combination in 11 of the 15 cross-validation folds (73.3%), while Q3, Q6, and Q9 were individually selected in 86.7%, 86.7%, and 80.0% of folds, respectively. Thus, although the selected self-report dimensions showed relatively high stability, the substantially lower cross-validated performance indicates that the model should be regarded as hypothesis-generating and proof-of-concept rather than as a validated predictive model. Full details of the internal validation are provided in Supplementary S3 (Section S3.4) of the Online Supplemental Materials.

## 4. Discussion

UHL is a prevalent condition, affecting approximately 7.2% of adults in a population-based US sample [1]. While traditional audiological evaluations such as pure-tone audiometry provide foundational diagnostic insights, they fail to capture the broader functional, psychological, and social challenges faced by individuals with UHL in real-world environments. The present study provides a multidimensional characterisation of the functional impact of unilateral hearing loss using an ecologically oriented laboratory paradigm. Rather than identifying deficits in a single domain, the findings indicate that UHL is associated with differences across multiple complementary aspects of everyday communication, including speech understanding, subjective hearing difficulties, social participation, perceived listening demand and fatigue, and distinct head-orientation behaviour. Together, these results highlight that functional hearing difficulties in UHL are inherently multidimensional and are therefore unlikely to be adequately captured by conventional audiological measures alone.

A significant strength of this study lies in its ecologically oriented design. The National Acoustic Laboratories Dynamic Conversations Test (NAL-DCT) was employed to evaluate speech-in-noise comprehension in a dynamic laboratory listening environment designed to approximate important features of everyday communication. Compared with traditional clinical speech testing, this paradigm incorporates several characteristics of complex everyday listening, including continuous conversation, multiple talkers, spatially distributed speech sources, and realistic background noise. Additionally, head-movement tracking provides a novel perspective on head-orientation behaviour in individuals with UHL during communication. The integration of these methods increases the relevance of the laboratory findings to everyday listening while retaining experimental control.

The results revealed that UHL participants face significant challenges across multiple domains, as shown by their performance on the SSQ12 and SPaRQ questionnaires. The SSQ12 highlighted significant difficulties in speech understanding, spatial hearing, and qualities of hearing, consistent with previous findings using the longer SSQ19 questionnaire in UHL populations [5]. Furthermore, the UHL group in this study reported lower SSQ12 global scores (mean = 4.31) than those reported by Dalla Costa et al. [26] for individuals with mild-to-severe unilateral hearing loss using hearing aids (mean = 7.53). Although differences in sample characteristics and study design preclude direct comparison, this observation highlights the need for studies directly evaluating how amplification influences perceived functional hearing in individuals with UHL. To our knowledge, this is the first study to apply the SPaRQ questionnaire to a UHL population, providing novel insights into the social and psychological impacts of this group. The findings align with prior research that has shown that UHL negatively affects quality of life, leading to increased social withdrawal, anxiety, and reduced self-esteem [8,9]. These findings are also consistent with the recent study by Apa et al. [10], who reported poorer social functioning and psychological well-being in working adults with SSD than in normal-hearing controls. Although different instruments were used, their social-functioning findings converge with the greater social participation restrictions observed with the SPaRQ in the present study.

Speech-in-noise performance was modestly poorer in the UHL group. In the primary analysis, NAL-DCT scores were estimated to be 6.08 percentage points lower in participants with UHL than in those with NH, and this difference was statistically significant. Importantly, the age-adjusted sensitivity analysis yielded a similar estimated between-group difference of 5.58 percentage points, although the effect no longer reached statistical significance. Age itself was not significantly associated with NAL-DCT performance. Thus, while the magnitude of the observed group difference was relatively stable after adjustment for age, its statistical evidence was less robust and should be interpreted cautiously given the modest sample size and the between-group age imbalance. The modest reduction in speech-in-noise performance observed in the UHL group is consistent with numerous previous studies demonstrating impaired speech perception in complex listening environments among adults with unilateral hearing loss [2,4,5,6]. This finding suggests that speech recognition scores alone may underestimate the functional consequences of unilateral hearing loss, particularly when listeners adopt compensatory strategies that help preserve communication performance. Consistent with this interpretation, the present study identified concurrent differences in perceived listening demand, mental fatigue, and head-orientation behaviour, together with descriptively longer reaction times in participants with UHL. Although the between-group difference in reaction time was not statistically significant, these complementary findings support the value of a multidimensional assessment of functional hearing that extends beyond speech-recognition accuracy alone.

The auditory reaction-time task showed that responses were significantly slower in background noise than in quiet, with model-estimated reaction times approximately 17% longer in noise. Although reaction times were descriptively longer in participants with UHL than in those with NH, with an estimated between-group difference of approximately 11%, this effect did not reach statistical significance. The robust effect of background noise is consistent with previous studies showing that adverse listening conditions can increase reaction times or interfere with behavioural performance even when speech intelligibility is maintained [27]. Reaction time is not a process-specific measure of listening effort and can be influenced by several factors, including auditory perception, sustained attention, decision-making, general processing speed, and motor response [28,29]. Accordingly, the present RT findings provide evidence that auditory response speed was sensitive to adverse listening conditions, but do not provide sufficient evidence for a UHL-specific slowing of reaction time or for greater listening effort in the UHL group. This distinction is important despite previous patient-reported evidence showing that adults with UHL experience increased listening effort and communication difficulties in complex auditory environments [4]. In the present study, greater perceived listening demand and mental fatigue were evident in the self-reported measures, but the specific cognitive mechanisms underlying these experiences cannot be determined from the current paradigm.

Head-orientation tracking during the NAL-DCT showed that participants with UHL adopted a significantly more negative mean head orientation, towards the poorer-hearing side, and exhibited greater within-participant orientation variability than NH participants. One possible acoustic explanation is suggested by the experimental geometry. At the mean orientation observed in the UHL group (approximately $-38^{\circ }$), the talker located at $-45^{\circ }$ would be positioned almost directly in front of the listener, whereas the talker at $+22.5^{\circ }$ would remain on the better-hearing side. Such an orientation could reduce the head-shadow disadvantage for speech originating from the poorer-hearing side while maintaining favourable acoustic access to the other talker at the better ear [30]. However, the present study did not directly test whether this orientation improved speech intelligibility. Importantly, these findings should be interpreted within the specific experimental configuration. The asymmetric loudspeaker arrangement and the LEDs indicating the active talker may themselves have influenced head orientation, while the laboratory setup lacked natural visual cues such as lip movements and facial expressions and removed some of the social constraints present in face-to-face communication. Participants with UHL also frequently returned to a $0^{\circ }$ azimuth position, possibly because this was the setup’s initial position and may have been perceived as the default orientation for recording responses between conversations. Although the observed head-orientation behaviours may be consistent with an adaptive compensatory response under this specific experimental configuration, the present study does not establish that they represent a generalised real-world strategy, were consciously adopted, or directly improved speech understanding. Future studies experimentally manipulating head orientation will be important to determine their functional role.

The exploratory regression analysis identified a combination of reaction time in noise and only three targeted self-report items that showed a strong apparent in-sample fit within the UHL sample (adjusted $R^{2}$ = 0.71). However, this value represents apparent in-sample fit following data-driven model selection. Internal validation of the complete model-selection procedure using leave-one-out cross-validation yielded substantially lower predictive performance ($R_{\mathrm{CV}}^{2}$ = 0.19), indicating considerable optimism in the apparent fit. Nevertheless, the selected self-report dimensions showed relatively high stability across cross-validation folds, suggesting that these variables may represent potentially informative dimensions for further investigation. The similar standardised associations of reaction time and the composite self-report score with NAL-DCT performance further illustrate the potential value of combining behavioural and subjective information. Taken together, these findings provide a hypothesis-generating proof-of-concept that inter-individual variability in functional speech-in-noise performance may be captured using a brief auditory task together with a small number of targeted questions. Replication and external validation in larger independent cohorts will be required before defining screening thresholds, estimating individual performance, or considering implementation in clinical or mobile assessment platforms.

The findings may also have implications for future research on rehabilitation. Currently, only 2% of individuals with UHL use hearing aids or assistive devices [1], highlighting a substantial gap in service uptake. By demonstrating the multifaceted challenges associated with UHL—including speech-in-noise difficulties, spatial-hearing difficulties, greater self-reported listening demands, and head-orientation behaviours—the present findings identify several functional domains that could be considered in future studies of rehabilitation. For example, future research could investigate whether auditory-cognitive or communication training can support listening in complex environments, whether spatial-hearing training or head-orientation strategies provide functional benefit, and whether social-support approaches can help address the psychosocial consequences of UHL. Similarly, future studies could examine how these functional outcomes respond to non-device strategies, environmental modifications, or established device-based rehabilitation options such as hearing aids, CROS systems, and cochlear implants. Importantly, the present study did not evaluate rehabilitation outcomes or compare treatment modalities, and therefore does not provide evidence for the effectiveness of any particular intervention. Rather, the multidimensional differences identified here may help define relevant outcomes and hypotheses for future rehabilitation studies.

Taken together, these findings support the use of multimodal assessment strategies that integrate behavioural performance, self-reported outcomes, and head-orientation behaviour to achieve a more comprehensive evaluation of functional hearing difficulties associated with UHL.

**Study limitations**

The present findings should be interpreted in light of several limitations. First, the relatively small sample size limited the statistical power to examine important sources of variability within the UHL cohort. Although all participants met the study definition of unilateral sensorineural hearing loss, the cohort was heterogeneous in terms of hearing-loss severity, age at onset, and duration. It included individuals with moderate, severe, and profound hearing loss in the poorer ear, with only a small number of participants representing the moderate and severe ranges, as well as individuals with congenital or early-acquired UHL and others with adult-onset UHL. Because 11 of the 15 participants had profound UHL, the cohort predominantly represented the more severe end of the UHL spectrum. Consequently, generalisation of the present findings to individuals with moderate or severe UHL should be made cautiously. Duration of UHL at the time of testing consequently ranged from approximately 1 to 65 years. These differences may influence residual audibility and access to binaural cues, as well as the development of auditory adaptation and compensatory listening behaviours. In particular, age at onset and duration of UHL may have contributed to inter-individual variability in head-orientation behaviour, self-reported difficulties, and speech-in-noise performance. Given the small sample, the study was not sufficiently powered to determine whether these outcomes varied reliably according to hearing-loss severity, age at onset, or duration. The findings should therefore be interpreted as describing the UHL group as a whole rather than providing reliable estimates for specific UHL subgroups.

In addition, participants in the UHL group were significantly older than those in the NH group. To examine the potential influence of this imbalance, age-adjusted sensitivity analyses were conducted for auditory reaction time and speech-in-noise comprehension. Adjustment for age did not materially alter the reaction-time findings: the effect of listening condition remained significant, while neither hearing group nor age was significantly associated with reaction time. For NAL-DCT performance, adjustment for age produced only a modest change in the estimated UHL–NH difference (from 6.08 to 5.58 percentage points), and age itself was not significantly associated with performance. However, the hearing-group effect no longer reached statistical significance after age adjustment. Therefore, although the sensitivity analyses do not indicate that age accounted for the observed between-group differences, the speech-in-noise finding should be interpreted cautiously because its statistical significance was sensitive to age adjustment in this relatively small sample. Larger, age-matched and stratified cohorts will be required to better isolate the specific contribution of unilateral hearing loss and to determine how functional outcomes vary according to the severity of hearing loss in the poorer ear.

Finally, although leave-one-out cross-validation was used to quantify the optimism associated with the exploratory regression and its data-driven model-selection procedure, the very small UHL sample limits the precision of this internal validation; therefore, the model should be considered hypothesis-generating until replicated and externally validated in larger independent cohorts.

## 5. Conclusions

This study provides a multidimensional characterisation of the functional impact of unilateral hearing loss using an ecologically oriented assessment paradigm. Compared with adults with normal hearing, individuals with UHL showed greater self-perceived hearing difficulties, greater social participation restrictions, and different head-orientation behaviours under the experimental listening configuration. Speech-in-noise comprehension was modestly poorer in the UHL group in the primary analysis, although the hearing-group effect no longer reached statistical significance after adjustment for age. Reaction times were significantly longer in noise than in quiet across participants, whereas the estimated difference between the UHL and NH groups was not statistically significant.

Together, these findings highlight that the functional consequences of UHL extend beyond speech recognition alone and arise from the interaction of perceptual, behavioural, and self-perceived communication difficulties. Consequently, no single outcome measure is likely to fully capture the everyday impact of UHL, highlighting the value of integrating complementary objective and subjective assessments into functional hearing evaluations.

Finally, the exploratory regression analysis identified a simple combination of reaction time in noise and three selected post-task self-report items that showed strong apparent in-sample associations with speech-in-noise performance within the UHL group. However, the substantially lower performance obtained following internal cross-validation indicates considerable optimism in the apparent model fit. Accordingly, this hypothesis-generating, proof-of-concept analysis requires validation in larger independent cohorts before any clinical application, but highlights the potential value of combining accessible behavioural and self-reported measures in the future development of brief, personalised functional assessment approaches for individuals with UHL.

## Figures and Tables

**Table 1:** Fixed-effect coefficients from the generalised linear mixed-effects model of reaction times, with listening condition and hearing group as fixed effects and participant as a random intercept. The model employed a Gamma distribution with a log link and was fitted using the Laplace approximation. The intercept represents the normal-hearing (NH) group in the Noise condition. Number of observations: 375. SE: standard error; 95% CI: 95% confidence interval.

Fixed Effect	β	SE	95% CI	*p*-Value
(Intercept)	6.6642	0.0493	[6.5672, 6.7611]	<0.001
Quiet	−0.15774	0.0261	[−0.2090, −0.1064]	<0.001
UHL	0.1044	0.0698	[−0.0328, 0.2417]	0.136

**Table 2:** Fixed-effect coefficients from the linear mixed-effects model of NAL-DCT scores (% correct), with hearing group and conversation (Run) as fixed effects and participant as a random intercept. The intercept represents the estimated NAL-DCT score for the NH group in Run 1. Number of observations: 186. SE: standard error; 95% CI: 95% confidence interval.

Fixed Effect	β	SE	95% CI	*p*-Value
(Intercept)	81.33	3.00	[75.41, 87.25]	<0.001
Run 2	−25.81	3.35	[−32.41, −19.21]	<0.001
Run 3	1.29	3.35	[−5.31, 7.89]	0.700
Run 4	−3.87	3.35	[−10.47, 2.73]	0.249
Run 5	−0.32	3.35	[−6.92, 6.28]	0.923
Run 6	−16.77	3.35	[−23.38, −10.17]	<0.001
UHL	−6.08	2.99	[−11.98, −0.18]	0.043

**Table 3:** Estimated coefficients from the best-fitting exploratory linear regression model relating NAL-DCT scores to standardised reaction time in noise and the standardised mean score across three self-report items: noise interference (Q3), frustration (Q6), and missing the start of sentences (Q9).

Fixed Effect	β	SE	*t*-Stat	*p*-Value
(Intercept)	67.667	1.1756	57.562	<0.001
Reaction time	−5.9862	1.2622	−4.7426	0.00047814
Self-perceived (Q3, Q6, Q9)	−6.0944	1.2622	−4.8283	0.00041315

## Data Availability

All raw data, processed data, programming scripts, and other research materials supporting the findings of this study are available as Supplementary Materials in Supplementary S1–S3.

## Supplementary Materials

The following supporting information can be downloaded at https://www.mdpi.com/article/10.3390/audiolres16050133/s1. Supplementary S1: Demographics and hearing assessments. Supplementary S2: Figures, tables, and statistical analyses. Supplementary S3: Supporting analyses.

## Abbreviations

The following abbreviations are used in this manuscript:

UHL	Unilateral hearing loss
SSD	Single-sided deafness
NH	Normal hearing
SSQ12	Speech, Spatial and Qualities of Hearing Scale
SPaRQ	Social Participation Restrictions Questionnaire
NAL-DCT	National Acoustic Laboratories Dynamic Conversations Test
PTA	Pure-tone average
RT	Reaction time
GLME	Generalised linear mixed-effects
ARTE	Ambisonics Recordings of Typical Environments
IELTS	International English Language Testing System
SNR	Signal-to-noise ratio
LME	Linear mixed-effects
