# Supplementary material for: Unilateral Hearing Loss: Listening Ability, Social Participation and Adaptive Strategies to Challenges in the Real World
Source: Audiol Res. 2026 Sep 7;16(5):133. doi: 10.3390/audiolres16050133 (PMC13600114; doi:10.3390/audiolres16050133)
Supplement: Supplementary file 1 [file audiolres-16-00133-s001.zip › S3.pdf]

Unilateral Hearing Loss: Listening Ability, Social Participation and Adaptive Strategies to Challenges in the Real World

Joaquin T. Valderrama[1,2,3,4,*], Colin Barbier[3,5,6], Paola Incerti[3,4], Jorge Mejia[3,7], Melanie Ferguson[3,8]

1. *Department of Signal Theory, Telematics and Communications, University of Granada, 18014 Granada, Spain.*
2. *Research Centre for Information and Communications Technologies (CITIC-UGR), University of Granada, 18014 Granada, Spain.*
3. *National Acoustic Laboratories, Sydney, NSW 2113, Australia;* cquba@dtu.dk (C.B.); paola.incerti@nal.gov.au (P.I.); geogem245@gmail.com (J.M.); melanie.ferguson@curtin.edu.au (M.F.)
4. *Department of Linguistics, Macquarie University, Sydney, NSW 2113, Australia.*
5. *Hearing Systems, Department of Health Technology, Technical University of Denmark, 2800 Copenhagen, Denmark.*
6. *Copenhagen Hearing and Balance Center, Rigshospitalet, 2100 Copenhagen, Denmark.*
7. *School of Computing, Macquarie University, Sydney, NSW 2113, Australia.*
8. *Curtin School of Allied Health, Faculty of Health Sciences, Curtin University, Perth, WA 6102, Australia.*

* *Correspondence:* jvalderrama@ugr.es

Supplementary S3

Supporting analyses

This appendix provides supporting analyses conducted to assess the robustness and interpretation of the main findings. It includes multiple-comparisons correction and non-parametric sensitivity analyses of the self-report outcomes, age-adjusted analyses of auditory reaction time and speech-in-noise comprehension, additional analyses of head orientation and the influence of missing head-tracking recordings, and internal validation of the exploratory regression analysis. These analyses complement the primary analyses reported in the main manuscript and provide additional information regarding the robustness and uncertainty of the findings.

Contents

S3.1. Multiple-comparisons correction and sensitivity analyses of self-report outcomes 2

S3.1.1. Standardised questionnaires 2

S3.1.2. Post-task self-report items 2

S3.2. Age sensitivity analyses 3

S3.2.1. Auditory reaction time 3

S3.2.2. Speech-in-noise comprehension 3

S3.3. Head-orientation analyses and sensitivity to missing recordings 5

S3.3.1. Between-group differences in head orientation 5

S3.3.2. Sensitivity analysis for missing head-tracking recordings 5

S3.4. Internal validation of the exploratory regression analysis 7

S3.1. Multiple-comparisons correction and sensitivity analyses of self-report outcomes

Multiple between-group comparisons were performed for the self-report outcomes included in the study. To account for the increased risk of false-positive findings arising from these related comparisons, the Benjamini-Hochberg false discovery rate (FDR) procedure was applied separately within the standardised-questionnaire and post-task self-report families. The analyses of the established SSQ12 and SPaRQ outcomes formed part of the planned multidimensional characterisation of UHL, whereas the item-level analyses of the 10 post-task self-report questions were exploratory.

S3.1.1. Standardised questionnaires

Between-group comparisons were performed for four SSQ12 outcomes—speech hearing, spatial hearing, qualities of hearing, and the global score—and two SPaRQ subscales—social behaviour and social perceptions. The six corresponding p-values were jointly adjusted using the Benjamini-Hochberg FDR procedure.

All six between-group differences remained statistically significant after FDR correction (all FDR-adjusted $p<0.001$; Table C1). Thus, correction for multiple comparisons did not alter the inferential pattern obtained for the standardised questionnaire outcomes.

Questionnaire	Outcome	Raw p-value	FDR-adjusted p-value
SSQ12	Speech hearing	2.03×10^{-8}	1.04×10^{-7}
SSQ12	Spatial hearing	2.73×10^{-7}	4.17×10^{-7}
SSQ12	Qualities of hearing	0.000452	0.000452
SSQ12	Global score	3.47×10^{-8}	1.04×10^{-7}
SPaRQ	Social behaviour	2.78×10^{-7}	4.17×10^{-7}
SPaRQ	Social perceptions	8.64×10^{-7}	1.04×10^{-6}

Table S3.1. Raw and Benjamini-Hochberg false discovery rate (FDR)-adjusted *p*-values for between-group comparisons of the SSQ12 and SPaRQ outcomes.

S3.1.2. Post-task self-report items

Between-group differences in the 10 post-task self-report items were initially assessed using independent-samples *t*-tests. Because these item-level analyses were exploratory and involved multiple related comparisons, the 10 corresponding *p*-values were jointly adjusted using the Benjamini-Hochberg false discovery rate (FDR) procedure. Following FDR correction, nine of the 10 items remained statistically significant, with only Q1 (perceived noisiness) remaining non-significant. Thus, correction for multiple comparisons did not alter the original pattern of statistical significance.

Given the five-level ordinal response format and the relatively small sample size, we additionally performed a non-parametric sensitivity analysis using Wilcoxon rank-sum tests. The 10 resulting *p*-values were again adjusted using the Benjamini-Hochberg FDR procedure. The non-parametric analysis yielded the same inferential pattern as the primary analysis: Q2–Q10 remained statistically significant after FDR correction, whereas Q1 remained non-significant. These findings indicate that the item-level conclusions were robust both to correction for multiple comparisons and to the use of a non-parametric test that does not rely on the distributional assumptions of the independent-samples *t*-test.

Item	*t*-test raw *p*	*t*-test FDR *p*	Rank-sum raw *p*	Rank-sum FDR *p*
Q1	0.0634	0.0634	0.0678	0.0678
Q2	0.00177	0.00354	0.00180	0.00360
Q3	0.00492	0.00615	0.00975	0.0122
Q4	$3.86\times10{-5}$	$1.43\times10{-4}$	0.000320	0.00107
Q5	0.00430	0.00615	0.00505	0.00842
Q6	0.00457	0.00615	0.00753	0.0108
Q7	$5.71\times10{-5}$	$1.43\times10{-4}$	0.000456	0.00114
Q8	0.0180	0.0200	0.0242	0.0269
Q9	$4.84\times10{-5}$	$1.43\times10{-4}$	0.000229	0.00107
Q10	$3.10\times10{-5}$	$1.43\times10{-4}$	0.000108	0.00107

Table S3.2. Raw and Benjamini-Hochberg false discovery rate (FDR)-adjusted p-values from the primary independent-samples t-tests and the non-parametric Wilcoxon rank-sum sensitivity analyses of the 10 post-task self-report items.

S3.2. Age sensitivity analyses

Participants with unilateral hearing loss were significantly older than participants with normal hearing. Because age may influence auditory processing, behavioural response speed, and speech-in-noise performance, additional sensitivity analyses were conducted to examine whether the main between-group findings were robust to statistical adjustment for age.

These analyses were intended as complementary sensitivity analyses rather than replacements for the primary analyses reported in the main manuscript. Accordingly, the original statistical models were retained and age was introduced as an additional covariate, allowing us to assess whether the estimated effects of hearing group and listening condition were materially altered after accounting for between-participant differences in age.

Sections C2.1 and C2.2 present age-adjusted analyses for the two outcomes considered most likely to be influenced by age: auditory reaction time and speech-in-noise comprehension measured with the National Acoustic Laboratories Dynamic Conversations Test (NAL-DCT). For each outcome, we report the age-adjusted model specification, estimated coefficients, and corresponding statistical inference, and compare the resulting pattern with that obtained in the primary analysis.

S3.2.1. Auditory reaction time

To assess whether the between-group age difference influenced the reaction-time findings, the primary generalised linear mixed-effects model was repeated including mean-centred age as an additional fixed effect. The model retained the same Gamma distribution, log link function, participant-specific random intercept, and Laplace approximation used in the primary analysis.

The age-adjusted model showed a significant effect of listening condition, with reaction times remaining longer in noise than in quiet. The effect of hearing group remained non-significant, and age was not significantly associated with reaction time. Thus, adjustment for age did not materially alter the pattern of findings observed in the primary analysis.

Fixed effect	β	SE	95% CI	p-value
(Intercept)	6.6558	0.0509	[6.5556, 6.7559]	<0.001
Quiet	-0.1580	0.0261	[-0.2093, -0.1066]	<0.001
UHL	0.1218	0.0750	[-0.0257, 0.2692]	0.105
Age	-0.00124	0.00204	[-0.00525, 0.00277]	0.543

Table S3.3. Age was mean-centred before inclusion in the model. The intercept therefore represents the estimated log reaction time for a normal-hearing participant of mean age in the Noise condition. Coefficients are reported on the log scale.

S3.2.2. Speech-in-noise comprehension

To assess whether the between-group age difference influenced speech-in-noise comprehension, the primary linear mixed-effects model of NAL-DCT scores was repeated with mean-centred age included as an additional fixed effect. The model retained hearing group and conversation (Run 1–6) as fixed effects and participant as a random intercept, with conversation treated as a categorical factor to account for systematic differences in difficulty across the six NAL-DCT conversations.

Adjustment for age produced only a modest change in the estimated hearing-group difference. NAL-DCT scores were estimated to be 5.58 percentage points lower in participants with UHL than in those with NH, compared with 6.08 percentage points in the primary analysis. However, the hearing-group effect no longer reached statistical significance after adjustment for age ($\beta=-5.58$, SE = 3.27, 95% CI [-12.02, 0.87], $p=0.089$). Age itself was not significantly associated with NAL-DCT performance ($\beta=-0.037$, SE = 0.088, 95% CI [-0.211, 0.137], $p=0.676$). The overall effect of conversation remained highly significant ($F(5,178)=22.14$, $p<0.001$).

Thus, although adjustment for age did not substantially alter the magnitude of the estimated difference in speech-in-noise performance between the UHL and NH groups, it increased the uncertainty surrounding this estimate, such that the between-group effect no longer met the conventional threshold for statistical

significance. These findings indicate that the primary NAL-DCT group effect should be interpreted cautiously in view of the age imbalance between groups and the relatively small sample size.

Fixed effect	β	SE	95% CI	p-value
(Intercept)	81.09	3.08	[75.02, 87.15]	<0.001
Run 2	−25.81	3.35	[−32.41, −19.21]	<0.001
Run 3	1.29	3.35	[−5.31, 7.89]	0.700
Run 4	−3.87	3.35	[−10.47, 2.73]	0.249
Run 5	−0.32	3.35	[−6.92, 6.28]	0.923
Run 6	−16.77	3.35	[−23.38, −10.17]	<0.001
UHL	−5.58	3.27	[−12.02, 0.87]	0.089
Age	−0.037	0.088	[−0.211, 0.137]	0.676

Table S3.4. Age-adjusted linear mixed-effects model of NAL-DCT scores (% correct), with hearing group, conversation (Run), and age as fixed effects and participant as a random intercept. Run 1 and the normal-hearing (NH) group were the reference categories. Age was mean-centred before inclusion in the model. The intercept therefore represents the estimated NAL-DCT score for a normal-hearing participant of mean age in Run 1. SE: standard error; 95% CI: 95% confidence interval.

S3.3. Head-orientation analyses and sensitivity to missing recordings

Head orientation was recorded during each of the six NAL-DCT conversations, yielding 186 potentially available recordings across the 31 participants included in the study. Thirty-eight recordings (20.4%) were excluded because of technical or calibration problems, leaving 148 recordings for analysis. Of the excluded recordings, 23 were from the NH group (23/96; 24.0%) and 15 from the UHL group (15/90; 16.7%).

Missing recordings were not uniformly distributed across participants or conversations. Twelve participants had all six recordings available (5 NH and 7 UHL), 12 had five valid recordings (6 NH and 6 UHL), one NH participant had four valid recordings, and four participants had three valid recordings (3 NH and 1 UHL). One participant from each group had no valid head-tracking recordings and therefore did not contribute to the head-orientation analyses. No minimum number of valid recordings was specified *a priori*; participant-level estimates were calculated from all available recordings. In practice, all participants contributing to the analysis had at least three valid recordings, resulting in 15 NH and 14 UHL participants with analysable head-tracking data.

The excluded recordings were also distributed across all six NAL-DCT conversations, with 12, 6, 7, 8, 3, and 2 recordings excluded from Runs 1–6, respectively. Thus, 25 recordings were excluded from Set 1 (Runs 1–3) and 13 from Set 2 (Runs 4–6). Although exclusion was attributable to technical or calibration problems rather than participant performance, the uneven distribution of missing recordings means that a systematic influence on the head-orientation estimates cannot be excluded solely from the pattern of missingness. We therefore performed a sensitivity analysis restricted to participants with at least five of the six recordings available.

S3.3.1. Between-group differences in head orientation

For each participant, mean head orientation was calculated by averaging the mean orientations obtained across all usable NAL-DCT recordings. Negative values indicate orientation towards the poorer-hearing side, whereas positive values indicate orientation towards the better-hearing side. Within-participant head-orientation variability was quantified by calculating the standard deviation of head orientation within each usable recording and then averaging these standard deviations across recordings for each participant.

To avoid assuming equal between-group variances, Welch's independent-samples t-tests were used to compare the NH and UHL groups. Mean head orientation was −4.30° ± 3.72° in the NH group and −38.39° ± 18.55° in the UHL group. Participants with UHL therefore showed a significantly more negative mean head orientation than participants with NH (UHL−NH difference = −34.09°, 95% CI [−44.93°, −23.26°], Welch's $t(13.97) = -6.75$, $p < 0.001$), indicating greater orientation towards the poorer-hearing side under the experimental configuration.

Within-participant head-orientation variability was also significantly greater in the UHL group (21.14° ± 4.22°) than in the NH group (10.43° ± 5.12°; UHL−NH difference = 10.71°, 95% CI [7.14°, 14.28°], Welch's $t(26.62) = 6.16$, $p < 0.001$). Thus, participants with UHL differed from NH participants both in their average head orientation and in the range of orientations adopted during the conversations.

S3.3.2. Sensitivity analysis for missing head-tracking recordings

To examine whether the between-group findings were influenced by participants with substantial missing head-tracking data, the analyses were repeated after restricting the sample to participants with at least five of the six NAL-DCT recordings available. This criterion retained 11 NH and 13 UHL participants.

The between-group difference in mean head orientation was essentially unchanged. Mean orientation was −4.29° ± 3.95° in the NH group and −40.04° ± 18.21° in the UHL group, corresponding to a UHL−NH difference of −35.74° (95% CI [−46.93°, −24.56°], $p < 0.001$). Similarly, within-participant orientation variability remained greater in the UHL group (21.51° ± 4.15°) than in the NH group (9.50° ± 4.15°), with a between-group difference of 12.01° (95% CI [8.48°, 15.55°], $p < 0.001$).

Thus, restricting the analysis to participants with near-complete head-tracking data did not attenuate either between-group effect. The magnitude and direction of both the mean-orientation difference and the difference in within-participant variability were highly similar to those obtained in the primary analysis, providing no indication that the findings were driven by participants with substantial missing head-tracking data.

**Analysis**	**Outcome**	**NH mean ± SD**	**UHL mean ± SD**	**UHL – NH difference [95% CI]**	***p*-value**
**Primary** (15 NH, 14 UHL)	Mean orientation (°)	−4.30 ± 3.72	−38.39 ± 18.55	−34.09 [−44.93, −23.26]	<0.001
	Orientation variability (°)	10.43 ± 5.12	21.14 ± 4.22	10.71 [7.14, 14.28]	<0.001
**Sensitivity** ≥ 5/6 recordings (11 NH, 13 UHL)	Mean orientation (°)	−4.29 ± 3.95	−40.04 ± 18.21	−35.74 [−46.93, −24.56]	<0.001
	Orientation variability (°)	9.50 ± 4.15	21.51 ± 4.15	12.01 [8.48, 15.55]	<0.001

Table S3.5. Primary and sensitivity analyses of head orientation during the NAL-DCT. The sensitivity analysis was restricted to participants with at least five of the six head-tracking recordings available. Negative mean-orientation values indicate orientation towards the poorer-hearing side. Orientation variability represents the participant-level mean of the within-recording standard deviations of head orientation. Between-group comparisons were performed using Welch's independent-samples t-tests. CI: confidence interval; NH: normal hearing; UHL: unilateral hearing loss.

S3.4. Internal validation of the exploratory regression analysis

The exploratory regression analysis evaluated 176 candidate models relating speech-in-noise comprehension, measured using the NAL-DCT, to auditory reaction time and self-reported hearing difficulties in the 15 participants with UHL. The candidate set comprised one model containing reaction time alone and 175 models containing reaction time together with a self-report composite derived from all possible combinations of one, two, or three items from the 10-item post-task questionnaire. For combinations containing more than one questionnaire item, responses were averaged to form a single self-report predictor. The number of questionnaire items included in the composite was restricted to a maximum of three to achieve a pragmatic balance between explanatory performance and parsimony. The model yielding the highest adjusted R^2 in the complete sample was selected as the best-fitting exploratory model.

Because the same sample was used both to select the best-performing questionnaire combination and to estimate its apparent model fit, the resulting in-sample performance may be optimistic. We therefore performed leave-one-out cross-validation (LOOCV) of the complete model-selection procedure. For each of the 15 folds, one participant was excluded and all 176 candidate models were re-evaluated using only the remaining 14 participants. The model with the highest adjusted R^2 in the training sample was then used to predict the NAL-DCT score of the held-out participant. Thus, each participant's prediction was obtained from a model for which that participant had contributed neither to predictor selection nor to model fitting.

Cross-validated performance was quantified from the 15 held-out predictions using the cross-validated coefficient of determination (R^2_{CV}), root-mean-square error (RMSE), and mean absolute error (MAE). R^2_{CV} was calculated as

$$R^2_{\mathrm{CV}} = 1 - \frac{\sum_{i=1}^{n}(y_i - \hat{y}_i)^2}{\sum_{i=1}^{n}(y_i - \bar{y})^2}$$

where y_i, $\hat{y}_i$ and $\bar{y}$ represent the observed, held-out predicted, and mean observed NAL-DCT scores, respectively.

The model selected using the complete sample, comprising reaction time and the mean self-report score across Q3 (noise interference), Q6 (frustration), and Q9 (missing the start of sentences), showed a strong apparent in-sample fit ($R^2 = 0.751$, adjusted $R^2 = 0.709$, RMSE = 4.55 percentage points). However, validation of the complete model-selection procedure yielded substantially lower predictive performance ($R^2_{\mathrm{CV}} = 0.191$, RMSE = 7.34 percentage points, MAE = 5.82 percentage points). This reduction indicates considerable optimism in the apparent in-sample performance of the selected model.

Nevertheless, the variables identified in the complete-sample analysis showed relatively high selection stability across the LOOCV folds. The Q3-Q6-Q9 composite was selected as the best-performing combination in 11 of the 15 training folds (73.3%). Considered individually, Q3 and Q6 were each selected in 13 of 15 folds (86.7%), while Q9 was selected in 12 of 15 folds (80.0%). These results suggest that the specific self-report dimensions identified in the original exploratory analysis were relatively stable to the removal of individual participants, although the substantially lower cross-validated performance confirms that the apparent in-sample performance considerably overestimates the predictive performance that might be expected for new observations. Accordingly, the regression analysis should be regarded as hypothesis-generating and proof-of-concept rather than as a validated predictive model.

Performance measure	Apparent in-sample performance	LOOCV performance
R^2	0.751	-
Adjusted R^2	0.709	-
R^2_{CV}	-	0.191
RMSE (percentage points)	4.55	7.34
MAE (percentage points)	-	5.82

Table S3.6. Apparent and leave-one-out cross-validated performance of the exploratory regression analysis. LOOCV repeated the complete model-selection procedure within each training fold.
