# Supplementary material for: Unilateral Hearing Loss: Listening Ability, Social Participation and Adaptive Strategies to Challenges in the Real World
Source: Audiol Res. 2026 Sep 7;16(5):133. doi: 10.3390/audiolres16050133 (PMC13600114; doi:10.3390/audiolres16050133)
Supplement: Supplementary file 1 [file audiolres-16-00133-s001.zip › S2/Figure 1/Figure1.pdf]

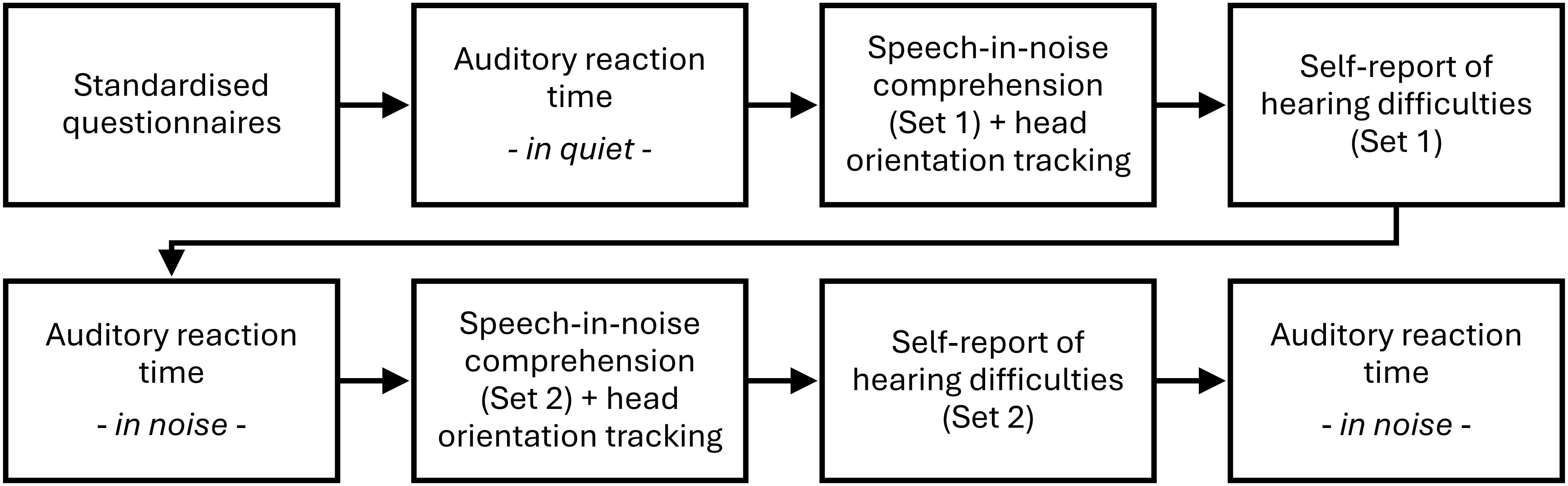
Standardised questionnaires
Auditory reaction time
- in quiet -
Speech-in-noise comprehension (Set 1) + head orientation tracking
Self-report of hearing difficulties (Set 1)
Auditory reaction time
- in noise -
Speech-in-noise comprehension (Set 2) + head orientation tracking
Self-report of hearing difficulties (Set 2)
Auditory reaction time
- in noise -
