# Supplementary material for: Unilateral Hearing Loss: Listening Ability, Social Participation and Adaptive Strategies to Challenges in the Real World
Source: Audiol Res. 2026 Sep 7;16(5):133. doi: 10.3390/audiolres16050133 (PMC13600114; doi:10.3390/audiolres16050133)
Supplement: Supplementary file 1 [file audiolres-16-00133-s001.zip › S2/Figure 2/Figure2.pdf]

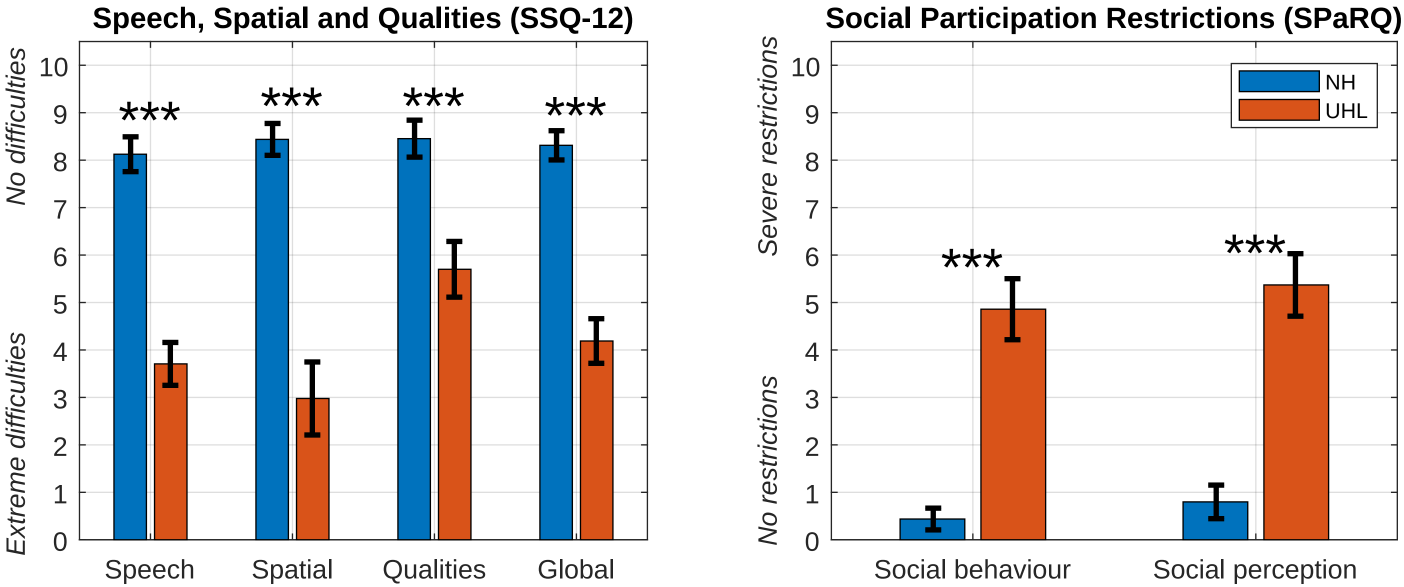

Speech, Spatial and Qualities (SSQ-12)
No difficulties
Extreme difficulties
10
9
8
7
6
5
4
3
2
1
0
***
***
***
***
Speech
Spatial
Qualities
Global
Social Participation Restrictions (SPaRQ)
Severe restrictions
No restrictions
NH
UHL
***
***
Social behaviour
Social perception
