# Supplementary material for: Unilateral Hearing Loss: Listening Ability, Social Participation and Adaptive Strategies to Challenges in the Real World
Source: Audiol Res. 2026 Sep 7;16(5):133. doi: 10.3390/audiolres16050133 (PMC13600114; doi:10.3390/audiolres16050133)
Supplement: Supplementary file 1 [file audiolres-16-00133-s001.zip › S2/Figure 3/Figure3.pdf]

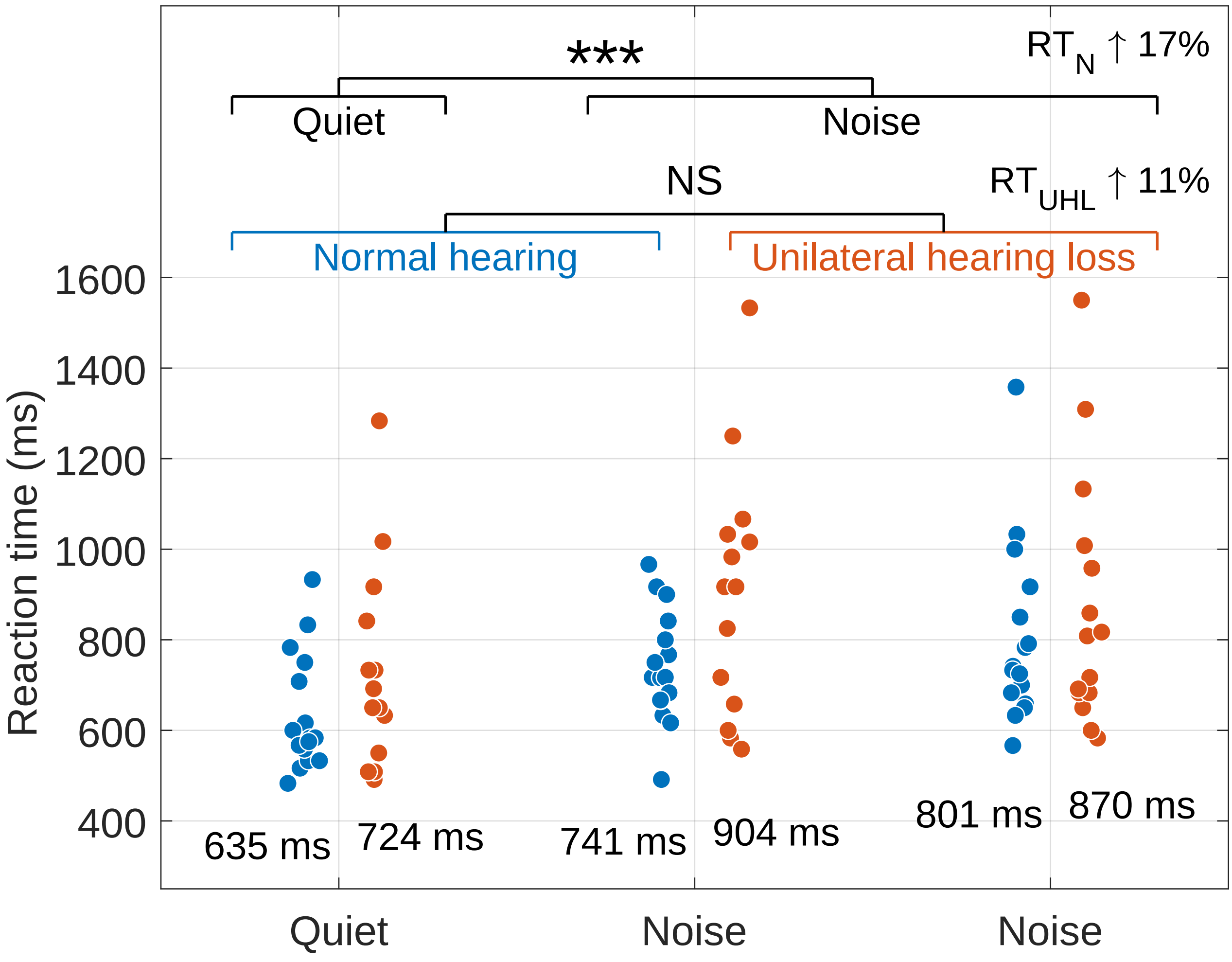

RT_N ↑ 17%
***
Quiet
Noise
NS
RT_UHL ↑ 11%
Normal hearing
Unilateral hearing loss
1600
1400
1200
1000
800
600
400
Reaction time (ms)
635 ms
724 ms
741 ms
904 ms
801 ms
870 ms
Quiet
Noise
Noise
