# Supplementary material for: Unilateral Hearing Loss: Listening Ability, Social Participation and Adaptive Strategies to Challenges in the Real World
Source: Audiol Res. 2026 Sep 7;16(5):133. doi: 10.3390/audiolres16050133 (PMC13600114; doi:10.3390/audiolres16050133)
Supplement: Supplementary file 1 [file audiolres-16-00133-s001.zip › S2/Figure 4/Figure4.pdf]

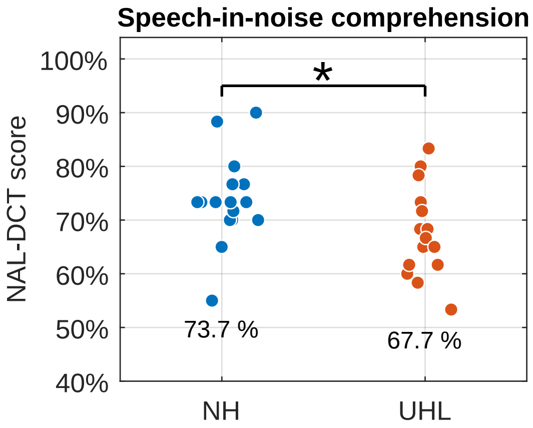

Speech-in-noise comprehension
NAL-DCT score
100%
90%
80%
70%
60%
50%
40%
*
73.7 %
67.7 %
NH
UHL
