# Supplementary material for: Unilateral Hearing Loss: Listening Ability, Social Participation and Adaptive Strategies to Challenges in the Real World
Source: Audiol Res. 2026 Sep 7;16(5):133. doi: 10.3390/audiolres16050133 (PMC13600114; doi:10.3390/audiolres16050133)
Supplement: Supplementary file 1 [file audiolres-16-00133-s001.zip › S2/Figure 5/Figure5.pdf]

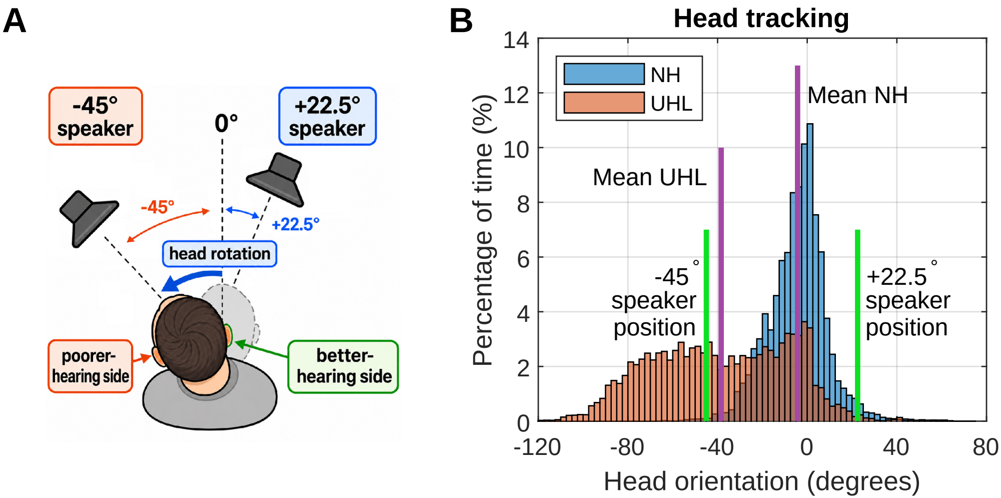
A
-45° speaker
0°
+22.5° speaker
-45°
+22.5°
head rotation
poorer-hearing side
better-hearing side
B
Head tracking
NH
UHL
Mean NH
Mean UHL
-45 ° speaker position
+22.5 ° speaker position
Percentage of time (%)
Head orientation (degrees)
0
2
4
6
8
10
12
14
-120
-80
-40
0
40
80
