# Supplementary material for: Unilateral Hearing Loss: Listening Ability, Social Participation and Adaptive Strategies to Challenges in the Real World
Source: Audiol Res. 2026 Sep 7;16(5):133. doi: 10.3390/audiolres16050133 (PMC13600114; doi:10.3390/audiolres16050133)
Supplement: Supplementary file 1 [file audiolres-16-00133-s001.zip › S2/Figure 6/Figure6.pdf]

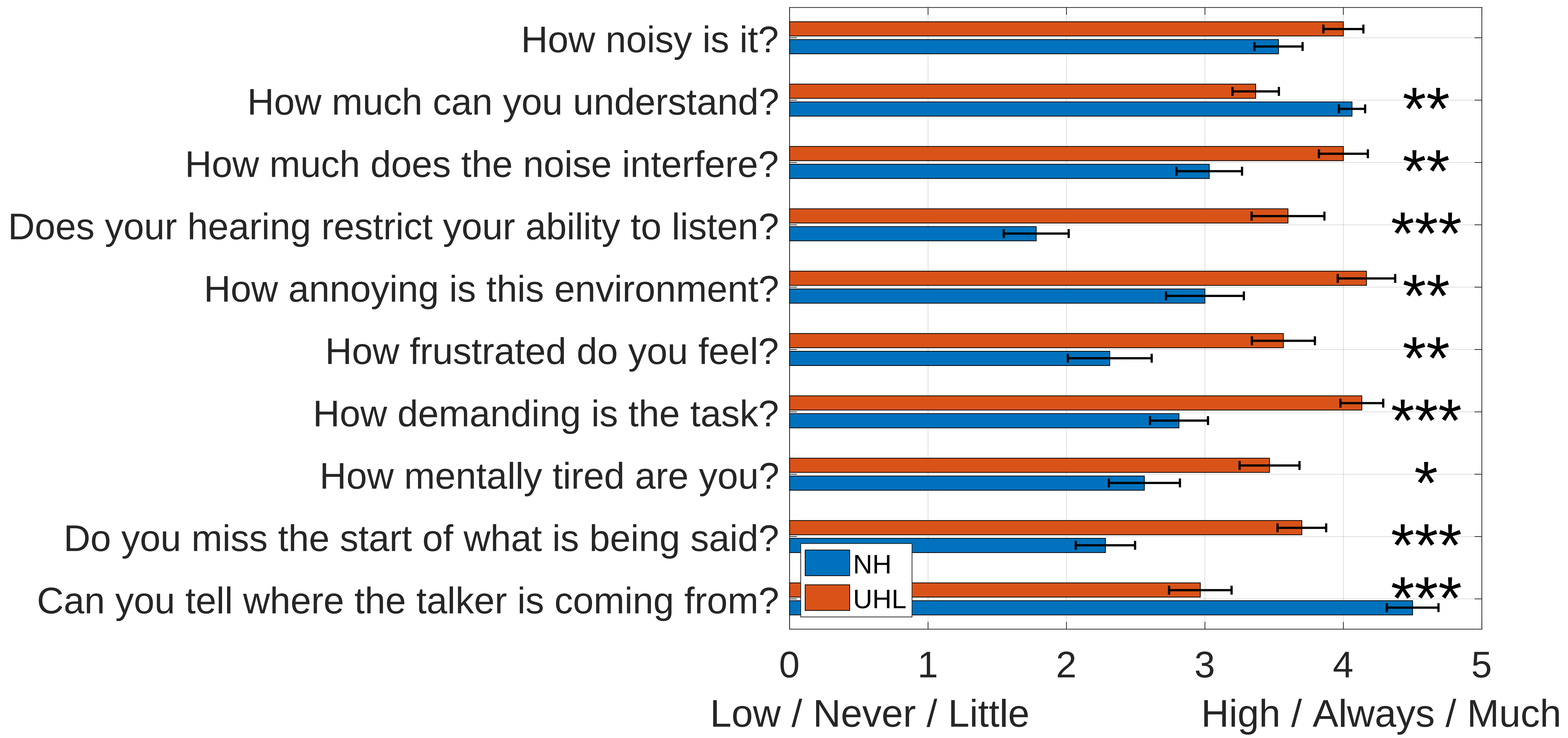
How noisy is it?
How much can you understand?
How much does the noise interfere?
Does your hearing restrict your ability to listen?
How annoying is this environment?
How frustrated do you feel?
How demanding is the task?
How mentally tired are you?
Do you miss the start of what is being said?
Can you tell where the talker is coming from?
**
**
***
**
**
***
*
***
***
NH
UHL
0
1
2
3
4
5
Low / Never / Little
High / Always / Much
