# Supplementary material for: Unilateral Hearing Loss: Listening Ability, Social Participation and Adaptive Strategies to Challenges in the Real World
Source: Audiol Res. 2026 Sep 7;16(5):133. doi: 10.3390/audiolres16050133 (PMC13600114; doi:10.3390/audiolres16050133)
Supplement: Supplementary file 1 [file audiolres-16-00133-s001.zip › S2/Figure 7/Figure7.pdf]

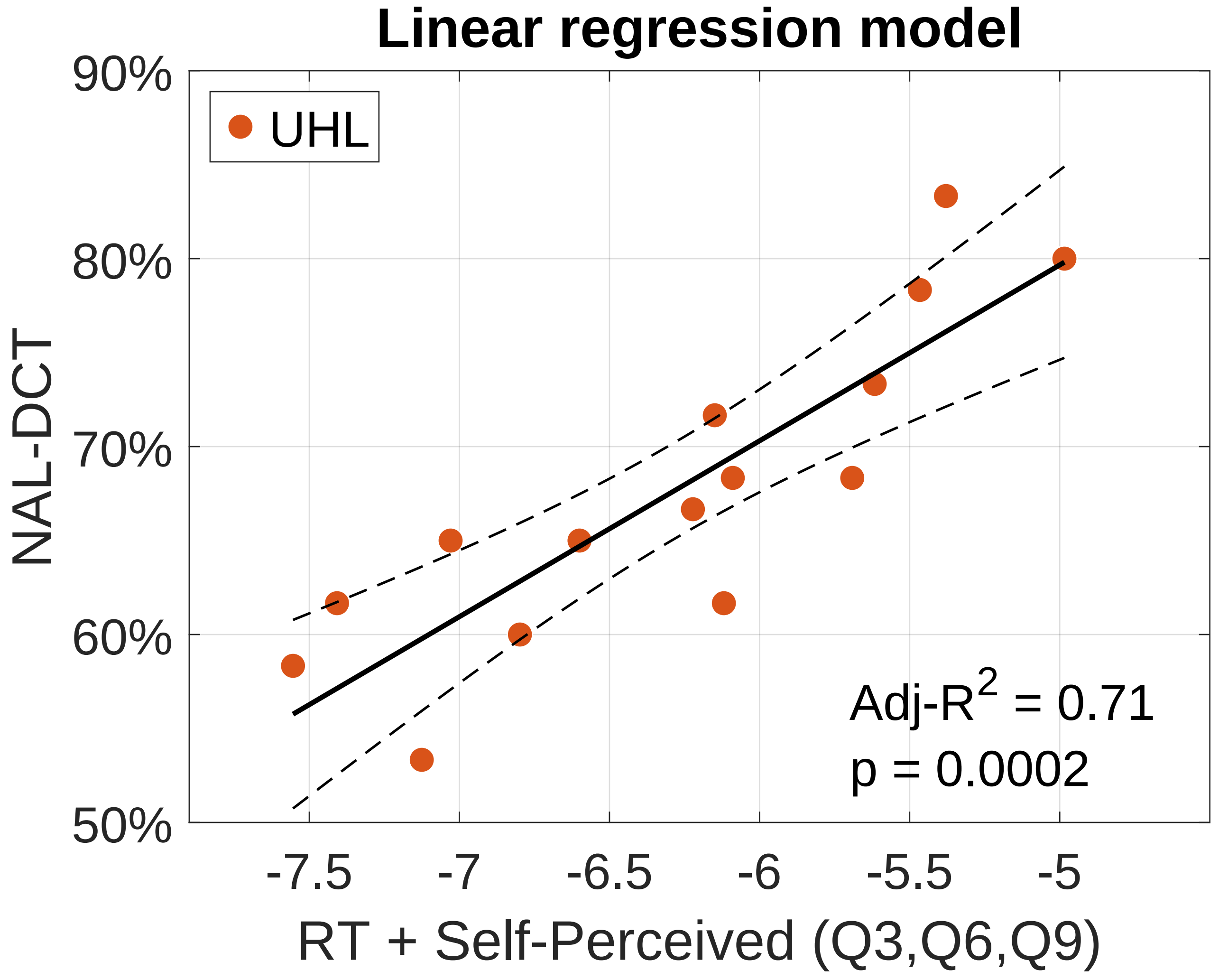

Linear regression model
90%
80%
70%
60%
50%
NAL-DCT
UHL
Adj-$R^2$ = 0.71
p = 0.0002
-7.5
-7
-6.5
-6
-5.5
-5
RT + Self-Perceived (Q3,Q6,Q9)
